# The Gene Desert Mammary Carcinoma Susceptibility Locus *Mcs1a* Regulates *Nr2f1* Modifying Mammary Epithelial Cell Differentiation and Proliferation

**DOI:** 10.1371/journal.pgen.1003549

**Published:** 2013-06-13

**Authors:** Bart M. G. Smits, Jill D. Haag, Anna I. Rissman, Deepak Sharma, Ann Tran, Alexi A. Schoenborn, Rachael C. Baird, Dan S. Peiffer, David Q. Leinweber, Matthew J. Muelbl, Amanda L. Meilahn, Mark R. Eichelberg, Ning Leng, Christina Kendziorski, Manorama C. John, Patricia A. Powers, Caroline M. Alexander, Michael N. Gould

**Affiliations:** 1 McArdle Laboratory for Cancer Research, Department of Oncology, University of Wisconsin School of Medicine and Public Health, Madison, Wisconsin, United States of America; 2 Department of Statistics, University of Wisconsin-Madison, Madison, Wisconsin, United States of America; 3 Department of Biostatistics and Medical Informatics, University of Wisconsin-Madison, Madison, Wisconsin, United States of America; 4 Department of Cell and Regenerative Biology, University of Wisconsin-Madison, Madison, Wisconsin, United States of America; National Cancer Institute, United States of America

## Abstract

Genome-wide association studies have revealed that many low-penetrance breast cancer susceptibility loci are located in non-protein coding genomic regions; however, few have been characterized. In a comparative genetics approach to model such loci in a rat breast cancer model, we previously identified the mammary carcinoma susceptibility locus *Mcs1a*. We now localize *Mcs1a* to a critical interval (277 Kb) within a gene desert. *Mcs1a* reduces mammary carcinoma multiplicity by 50% and acts in a mammary cell-autonomous manner. We developed a megadeletion mouse model, which lacks 535 Kb of sequence containing the *Mcs1a* ortholog. Global gene expression analysis by RNA-seq revealed that in the mouse mammary gland, the orphan nuclear receptor gene *Nr2f1/Coup-tf1* is regulated by *Mcs1a*. In resistant *Mcs1a* congenic rats, as compared with susceptible congenic control rats, we found *Nr2f1* transcript levels to be elevated in mammary gland, epithelial cells, and carcinoma samples. Chromatin looping over ∼820 Kb of sequence from the *Nr2f1* promoter to a strongly conserved element within the *Mcs1a* critical interval was identified. This element contains a 14 bp indel polymorphism that affects a human-rat-mouse conserved COUP-TF binding motif and is a functional *Mcs1a* candidate. In both the rat and mouse models, higher *Nr2f1* transcript levels are associated with higher abundance of luminal mammary epithelial cells. In both the mouse mammary gland and a human breast cancer global gene expression data set, we found *Nr2f1* transcript levels to be strongly anti-correlated to a gene cluster enriched in cell cycle-related genes. We queried 12 large publicly available human breast cancer gene expression studies and found that the median *NR2F1* transcript level is consistently lower in ‘triple-negative’ (ER-PR-HER2-) breast cancers as compared with ‘receptor-positive’ breast cancers. Our data suggest that the non-protein coding locus *Mcs1a* regulates *Nr2f1*, which is a candidate modifier of differentiation, proliferation, and mammary cancer risk.

## Author Summary

Most non-Mendelian disease variants identified through genome-wide association studies are low-penetrance, common in the population and located in non-protein coding genomic loci. It is currently unknown how these loci modulate disease risk. Insights in their mechanisms could lead to the development of novel prevention or early intervention strategies. We used comparative genetics to model such loci in a rat model for breast cancer susceptibility. For the *Mcs1a* locus presented in this paper, we describe its non-protein coding localization and the mechanism through which it affects mammary carcinoma susceptibility, involving transcriptional regulation of the orphan nuclear receptor gene *Nr2f1/Coup-tf1* and mammary epithelial cell proliferation/differentiation. In addition, we show that low *NR2F1* transcript levels are associated with upregulation of cell cycle-related genes and high histological grade (grade 3, poorly differentiated, highly proliferative) in human breast cancers, including triple-negative therapy-resistant breast cancers. Our findings highlight the orphan nuclear receptor NR2F1 as a novel target for breast cancer prevention and/or intervention strategies. Since COUP-TFs (including NR2F1) are nuclear hormone receptors, whose crystal structure suggests these are ligand controlled, identification of the ligand for NR2FI could provide a potential breast cancer therapeutic.

## Introduction

An important indicator for breast cancer risk is the family history, suggesting a strong genetic component in breast cancer susceptibility [1]. The heritable portion of a woman's risk to breast cancer consists of numerous risk-increasing and risk-decreasing alleles. Through familial linkage studies in the 1990s, deleterious mutations affecting the coding regions of well-known tumor suppressor genes, i.e. *BRCA1* and *BRCA2*, were found to associate with increased breast cancer risk [2], [3]. Such mutations are rare in the population. More recently, genome-wide association studies (GWAS) have been employed to discover association of common variants with breast cancer susceptibility. GWAS have proven to be successful at uncovering loci harboring low-penetrance breast cancer susceptibility variants [4], [5], [6], [7], [8], [9], [10], [11], [12], [13], [14]. In the future, the identification of these variants will likely impact population-based risk prediction [15], [16]. Many of the variants are located in non-protein coding areas of the genome, such as promoters, introns and intergenic areas with large genomic regions without known genes, called gene deserts. It is anticipated that these variants are involved in gene regulation as exemplified by breast cancer susceptibility-associated variant rs2981582 that is correlated with *FGFR2* transcript levels in breast cancer [17] and normal breast tissue [18]. For other non-protein coding breast cancer-associated variants, their spatiotemporal regulatory function and gene targets are largely undefined. Moreover, mechanisms underlying common breast cancer-associated variants on the level of the mammary gland tissue and mammary epithelial cells (MECs) are unknown.

MEC proliferation and differentiation are strongly interconnected processes for which ample evidence exists that these are involved in the development of breast cancer. Recently, flow cytometry-based approaches have yielded many markers that aid in the understanding of the hierarchical order of MEC differentiation [19]. For mouse mammary epithelial cells (MMECs), luminal and basal/myoepithelial populations were identified based on expression of heat stable antigen (HSA; CD24) and β1 integrin (CD29), or HSA and α6 integrin (CD49f). Specific cells of the basal lineage expressing high levels of CD29 or CD49f and high levels of CD24 were shown to harbor repopulating ability in single cell transplantation assays in mice, suggesting the presence of bipotential mammary stem/progenitor cell activity [20], [21]. Markers for lineage-specific progenitor cells have also been identified in the mouse, including CD61, which enriches for mouse luminal progenitors [22]. Later, additional markers for luminal progenitors have been identified, which include c-kit and ALDH [23], [24]. We have reported a flow cytometry-based approach to identifying the luminal and basal/myoepithelial cell lineages in the rat mammary gland [25]. In the early 1990s, clonogenic rat mammary epithelial cells (RMECs) were found to stain with peanut lectin [26], [27]. There is strong interest in the biology of mammary stem/progenitor cells as these are thought to be the target cells for tumorigenic transformation events, mainly because of their immortality and ability to sire many generations of daughter cells. Interestingly, human germ line mutation of *BRCA1* has been shown to stimulate luminal-to-basal tumor formation by affecting the luminal progenitor cell pool and luminal cell fate [28], [29], exemplifying a consequence of the involvement of breast cancer susceptibility variants in MEC differentiation.

It is currently unclear if other (e.g. low-penetrance, non-protein coding) breast cancer-associated variants affect MEC proliferation and differentiation. In order to model breast cancer susceptibility loci in a mammalian organism, we have conducted a rat-human comparative genetics approach. Upon initiation of this approach we selected the rat mammary carcinogenesis model, as the arising mammary carcinomas well reflect specific aspects of human breast adenocarcinoma, i.e. staged progression and ovarian hormone responsiveness. The advantage of the rat-human comparative genetics approach is that the availability of mammalian genetic model organisms aids in the dissection of the mechanisms underlying the susceptibility loci [30]. The rat mammary carcinoma resistance quantitative trait locus (QTL) *Mcs1* was identified in the backcross progeny of an intercross between the resistant Copenhagen (Cop) and susceptible Wistar-Furth (WF) parental inbred rat strains [31], [32]. Physical confirmation of this resistance locus was presented in a study using a congenic recombinant inbred line having a large portion of the original *Mcs1* QTL from the Cop strain introgressed onto the WF genetic background [33]. Congenic rats harboring a homozygous *Mcs1* Cop allele had a 85% reduction of 7,12-dimethylbenz(*a*)anthracene (DMBA)-induced mammary carcinoma multiplicity as compared with congenic control animals homozygous for the susceptible WF *Mcs1* allele. Testing various other congenic lines with smaller Cop *Mcs1* portions on the WF genetic background revealed that the initial *Mcs1* QTL harbors three modifier loci of mammary carcinoma susceptibility, namely *Mcs1a*, *Mcs1b* and *Mcs1c* [33]. In this study, we describe the congenic fine-mapping of the *Mcs1a* critical interval to a ∼277 Kb region entirely embedded within a large gene desert on rat chromosome *2*. Using a congenic rat mammary gland transplantation assay, we show that the *Mcs1a* locus controls DMBA-induced mammary carcinoma development in a mammary cell-autonomous manner. While the rat mammary carcinogenesis model has proven value to study certain aspects of breast cancer etiology, complex genome-engineering technology for the rat is still under development. Since *Mcs1a* shows good evolutionary conservation to human, mouse and other mammalian species, we describe the genetic engineering of a novel megadeletion (MD) model in the mouse. Homozygous MD mice lack a large piece of the gene desert including the region orthologous to the *Mcs1a* critical interval. Taking advantage of both rodent genetic model systems we found an effect of this non-protein coding locus on MEC proliferation/differentiation and identified the orphan nuclear factor *Nr2f1/Coup-tf1* as the *Mcs1a* target gene. To investigate its translational potential we analyzed *NR2F1* transcript levels in available global gene expression data for human breast cancers. We show the correlation of low *NR2F1* transcript levels with high-grade and discuss the implication of this finding for human breast cancer. Using this rat-mouse-human comparative genetics approach we identified *Nr2f1* as a novel gene target for the development of breast cancer prevention or therapeutic strategies.

## Results

### The *Mcs1a* critical interval is located in a gene desert

**Figure 1:**
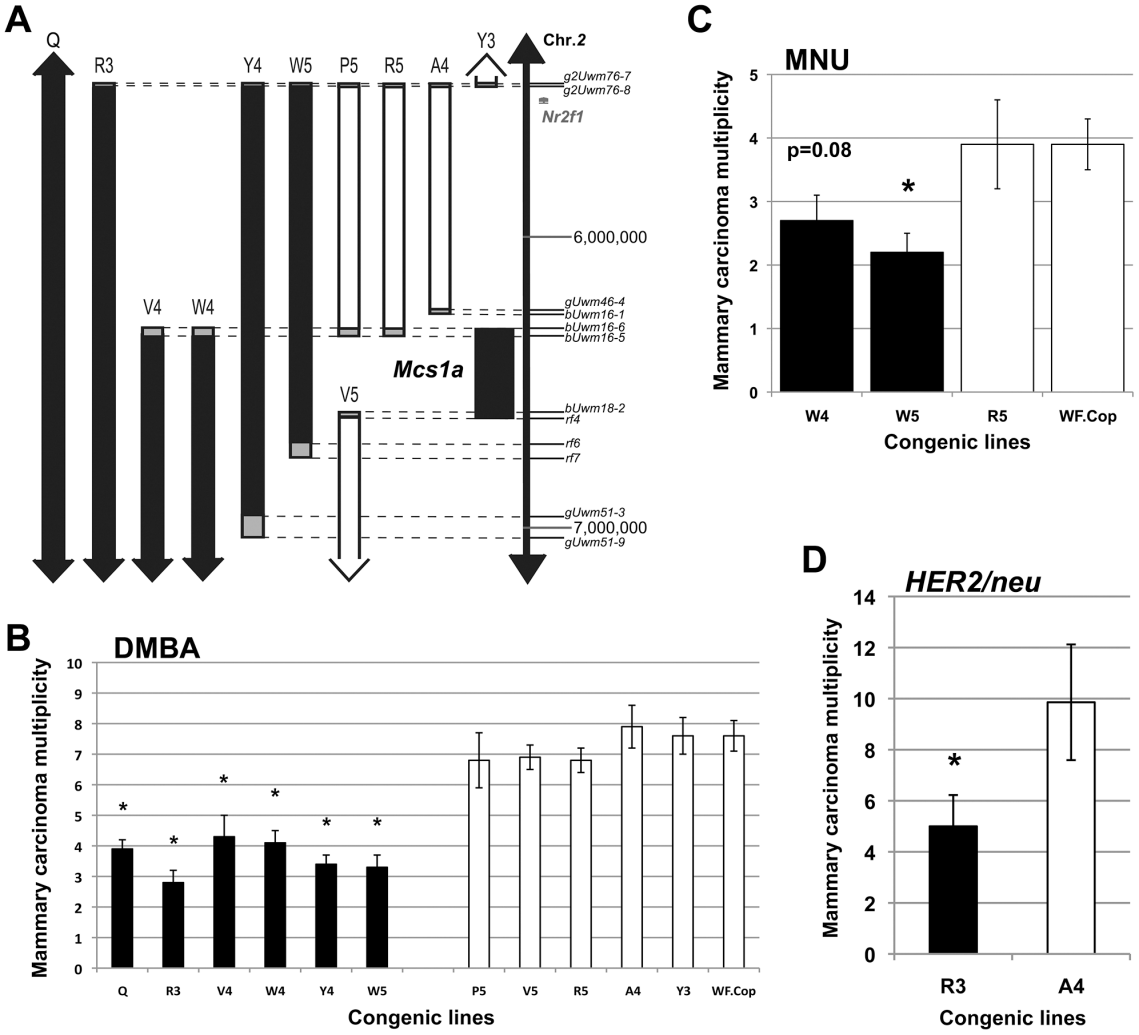
The rat mammary carcinoma susceptibility locus *1a* (*Mcs1a*) is located in a gene desert and confers resistance to three distinctly acting carcinogenic treatments. A) Genetic map of the congenic lines contributing to the positional identification of the *Mcs1a* locus on rat chromosome *2*. Each congenic line, as defined by genotyping the genetic markers indicated along the vertical scale bar (also listed in Table S1), represents a segment from the resistant Copenhagen (Cop) inbred strain introgressed into the susceptible Wistar-Furth (WF) genetic background. The critical interval for the *Mcs1a* resistance allele is defined by resistant congenic lines (filled bars) showing a *7,12*-dimethylbenz(a)anthracene (DMBA)-induced mammary carcinoma multiplicity that is lower than that of the susceptible congenic control line (WF.Cop), and susceptible congenic lines (open bars) showing a DMBA-induced mammary carcinoma multiplicity not different than that of the susceptible congenic control line. The grey boxes illustrate the areas of recombination. The coordinates (in bp) along the vertical axis are from the 2004 version of the rat genome (UCSC Genome Browser, rn4). B) DMBA-induced mammary carcinoma multiplicity phenotype for *Mcs1a* resistant congenic lines Q (n = 83), R3 (n = 24), V4 (n = 24), W4 (n = 28), Y4 (n = 45), and W5 (n = 41) and susceptible congenic lines P5 (n = 16), V5 (n = 56), R5 (n = 30), A4 (n = 24), Y3 (n = 38) and WF.Cop (n = 44). Congenic line Q originally defined the *Mcs1a* interval in our previous publication and is used as a reference for resistance [33]. C) *N*-methyl-*N*-nitrosourea (MNU)-induced mammary carcinoma multiplicity phenotype of resistant congenic lines W4 (n = 20) and W5 (n = 23), susceptible congenic line R5 (n = 14) and the susceptible congenic control line WF.Cop (n = 28). D) Mammary carcinoma multiplicity phenotype induced by mammary ductal infusion of retrovirus expressing the activated *HER2/neu* oncogene (*HER2/neu*) for resistant congenic line R3 (n = 15) and susceptible congenic line A4 (n = 14). In all graphs, resistant congenic lines are displayed as filled bars, susceptible congenic lines are displayed as open bars. Significant difference (P<0.05) from the susceptible congenic control line (panels B and C) or from susceptible congenic line A4 (panel D) is indicated by an asterisk.

We previously showed that the mammary carcinoma resistance allele *Mcs1a* from the Cop inbred strain when introgressed onto the susceptible WF inbred genetic background reduced DMBA-induced mammary carcinoma multiplicity by ∼50%, as compared with the susceptible congenic control line, not carrying the Cop *Mcs1a* allele [33]. Using multiple additional congenic lines, we now present further fine-mapping of the interval conferring the reduction in DMBA-induced mammary carcinoma multiplicity phenotype (Figure 1A). The resistant congenic lines have a significantly (P<0.001) lower mammary carcinoma multiplicity as compared with the susceptible congenic control line (WF.Cop; Figure 1B). The susceptible congenic lines have a mammary carcinoma multiplicity not different from the susceptible congenic control line (P>0.2). The resistant congenic lines together with the susceptible congenic lines V5 define the *Mcs1a* critical interval as a ∼277 Kb genomic region located in a gene desert on rat chromosome *2*. The gene desert is flanked by *Nr2f1* at the proximal side (Figure 1A) and *Arrdc3* at the distal side (outside the window in Figure 1A).

Using resistant congenic lines W4 and W5, susceptible congenic line R5 and the susceptible congenic control line WF.Cop, we tested if *Mcs1a* also confers resistance to *N*-methyl-*N*-nitrosourea (MNU)-induced mammary carcinogenesis. The resistant congenic line W5 showed a decreased mammary carcinoma multiplicity phenotype (P = 0.008) as compared with WF.Cop and the resistant congenic line W4 showed a strong trend (P = 0.08) towards a decreased MNU-induced mammary carcinoma multiplicity. The susceptible congenic line R5 was not different from the WF.Cop line (Figure 1C). Subsequently, carcinoma multiplicities following mammary ductal infusion of retrovirus expressing the activated *HER2/neu* oncogene were determined [34]. In this assay, the resistant congenic line R3 had a significantly reduced mammary carcinoma multiplicity (P = 0.04) as compared with the susceptible congenic line A4 (Figure 1D). These data show that Cop inbred strain-derived alleles of the *Mcs1a* locus (that include the smallest critical interval) introgressed on the susceptible genetic background confer resistance to three distinctly acting mammary carcinogenic treatments. These data suggest that the resistance mechanism likely manifests beyond the stage of (carcinogen-specific) cancer initiation.

### *Mcs1a* modulates susceptibility through a mammary cell-autonomous mechanism

To ask if *Mcs1a* acts via a mammary cell-autonomous mechanism, a mammary gland transplantation assay was carried out. Mammary gland tissue from donor animals of the susceptible inbred WF rats or the *Mcs1a* resistant congenic line Y4 was transplanted into the interscapular white fat pads of recipient animals with the same genotype or F1 animals of an intercross between WF and Y4. A total of 228 transplantations were performed, of which only 2 failed to produce a mammary outgrowth. For the 4 transplant groups (donor to recipient; susceptible to susceptible S:S, susceptible to F1 S:F1, resistant to F1 R:F1, and resistant to resistant R:R) carcinoma development following DMBA exposure was monitored (Table 1). The mammary carcinoma incidence at the transplant site was 41%, 36%, 13%, and 9% for transplant groups S:S, S:F1, R:F1, and R:R, respectively. Logistic regression analysis revealed that donor genotype (P = 0.0024), but not recipient genotype (P = 0.59) was significantly associated with transplant site carcinoma development (Table 1). The interaction between donor and recipient genotype was not significant (P = 0.44) for the dependent variable mammary gland transplant carcinoma susceptibility (Table 1). These data demonstrate that the mammary carcinoma susceptibility phenotype mediated by *Mcs1a* is transferable by transplantation of the mammary gland, indicating that *Mcs1a* modulates susceptibility in a mammary cell-autonomous manner.

**Table 1:** Mammary gland transplantation data and logistic regression analysis.

Mammary gland transplant data^1^						Logistic regression analysis^2^		
	S:S	S:F1	R:F1	R:R	Total	Dependent	Coefficient	P-value
0 carcinoma	48	48	33	30	159	Donor effect	1.9577	**0.0024** *
1 carcinoma	22	24	5	3	54	Recipient effect	0.4155	0.5907
2 carcinomas	12	3	0	0	15	Donor×recipient	−0.6460	0.4416
% transplant sites with 1+ carcinomas	41%	36%	13%	9%	30%			

1 Transplant groups (donor:recipient): S:S (susceptible:susceptible); S:F1 (susceptible:F1); R:F1 (resistant:F1); R:R (resistant:resistant). Susceptible = WF inbred strain; Resistant = resistant *Mcs1a* congenic line Y4; F1 = WFxY4 or Y4×WF.

2 Logistic regression was used to estimate the effect of donor and recipient and the interaction between donor and recipient for the dependent variable of mammary gland transplant carcinoma susceptibility (outcome = carcinoma presence or absence at transplant site) in susceptible WF and *Mcs1a* resistant congenic transplant groups.

* Statistically significant.

### A megadeletion (MD) mouse model for the rat *Mcs1a* locus reveals the orphan nuclear factor *Nr2f1* as a *Mcs1a* target gene

**Figure 2:**
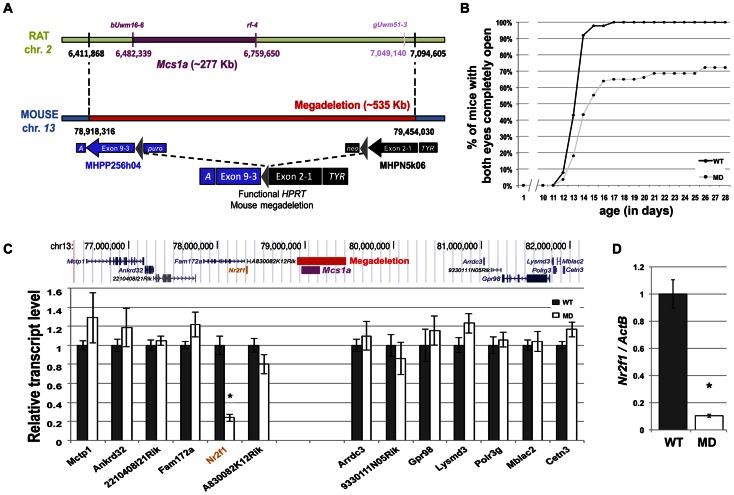
A megadeletion (MD) mouse model for the rat *Mcs1a* locus reveals *Nr2f1* as the *Mcs1a* target gene. A) Schematic representation of the rat *Mcs1a* locus and mouse orthologous locus that was deleted using mutagenic insertion and chromosome engineering resource (MICER) vector-assisted targeting. The horizontal bar, genetic markers and chromosomal base pair positions in purple represent the location of the *Mcs1a* critical interval on rat chromosome *2* (UCSC Genome Browser, rn4). The marker and base pair position in light purple delineate the location of the distal end of the *Mcs1a* critical interval at the time of design of the MICER project. The MD on mouse chromosome *13* is depicted as a red horizontal bar. The coordinates (in bp) are from the 2007 version of the mouse genome (UCSC Genome Browser, mm9). At the indicated base pair positions, a compatible pair of MICER vectors was placed, such that upon Cre-recombinase-driven excision in the embryonic stem-cell stage, a functional *Hypoxanthine-guanine phosphoribosyltransferase* (*HPRT*) gene was formed, which allowed for selection of a correctly targeted ES cell clone. Construct MHPP256h04 contained *Agouti* (*A*), *HPRT* exons 3–9, *loxP* (grey triangle) and a Puromycin resistance gene (*puro*); Construct MHPN5k06 contained a Neomycin resistance gene (*neo*), *loxP* (grey triangle), *HPRT* exons 1 and 2, and *Tyrosinase* (*TYR*). B) Graph showing the delayed eye-opening phenotype in MD mice (n = 83; FVB) as compared with WT littermates (n = 88; FVB). Plotted are on the vertical axis the percentage of mice in each genotype group with both eyes completely open and on the horizontal axis age in days. C) RNA-seq analysis of mammary gland gene expression from MD and WT control mice (n = 4 each; FVB). The transcript levels are shown as the average (+/− sem) counts from the RSEM algorithm output (used to estimate transcript levels [37]), normalized to the average of the WT group. Shown are transcripts within 2.5 Mb surrounding the gene desert. Only the transcript level of the orphan nuclear receptor gene *Nr2f1* was significantly different between MD and WT mice (P<0.05, indicated by an asterisk). D) Quantitative real-time PCR (Q-PCR) analysis verifying the differential transcript level of *Nr2f1* in mammary gland from MD and WT control mice (n = 12 each; FVB). The *Nr2f1* transcript level is shown relative to the transcript level of the *ActB* endogenous control gene.

Considering the evolutionary conserved nature of the locus, we sought to genetically engineer a mouse model for the rat *Mcs1a* locus. In mouse ES cells, we employed a MICER vector-assisted double targeting strategy to insert (on the same chromosome) *loxP* sites at either side of the gene desert region orthologous to the rat *Mcs1a* critical interval that was known at the time of design (Figure 2A). Both targeting steps were checked for proper integration of the MICER construct by Southern blot analysis. The proximally located MICER construct harbors the 3′ half (exons 3–9) of the *Hprt* gene and the distal construct harbors the 5′ half (exons 1–2) of *Hprt* that upon proper Cre-*lox* recombination form a functional *Hprt* gene. Following Cre-recombinase transfection and *Hprt* selection, the MD mutation was created and the mouse model was generated through blastocyst injections of karyotypically normal ES cells. After germ line transmission of the mutation, homozygous mutants were obtained and tested for lack of the 535 Kb targeted region in the *Mcs1a* orthologous gene desert on mouse chromosome *13* (Figure 2A). PCR tests using 4 different primer combinations within the deleted sequence and 2 primer combinations spanning the deletion showed consistent results that the region is indeed deleted. An example of a genotyping gel image is shown in Figure S1. The mutation was transferred through 10 generations of breeding to 2 inbred genetic backgrounds, namely FVB/N (FVB) and C57Bl/6 (B6).

Homozygous MD mice of both genetic backgrounds are viable and litter sizes are normal as compared with wild type (WT) animals, suggesting there is no embryonic or neonatal lethality associated with the mutation. Groups of homozygous MD and WT mice were monitored for obvious phenotypes. There was no difference in body weight up to 1 year of age and life span was not affected during the same period. An obvious phenotype we noticed was delayed eyelid opening of homozygous MD mice on both genetic backgrounds (Figure 2B, shown for FVB). While all WT animals had both eyelids completely open by 17 days of age, this was observed for only 65% of homozygous MD mice. For some animals, the closed eyelid phenotype persisted for months (unpublished data).

It is anticipated that a large portion of the non-protein coding capacity of the genome may be involved in the spatiotemporal regulation of gene expression [35]. For many non-coding elements, the target genes of regulation are unknown. We performed a global gene expression study by RNA-seq on mammary gland RNA samples from MD and WT mice (FVB). First, the quality-filtered reads were mapped to the mouse genome. We focused on reads mapping to the *Mcs1a*-associated gene desert to check for putative unknown transcripts located within the deleted region. We found only 6 reads from the WT samples aligning to the MD region, suggesting that no highly expressed unknown transcript exists within the region. As expected, no reads from the MD samples aligned to the deleted region.

Next, the reads were mapped to the mouse Ensembl reference set of 82,508 transcripts (annotated to 31,034 genes) using Bowtie [36]. Relative transcript abundance was determined using the RSEM algorithm [37]. For the detection of differential gene expression between MD and WT samples, the edgeR package was used [38]. First, we looked at the levels of transcripts within 2.5 Mb of either side of the *Mcs1a*-associated gene desert (Figure 2C). The only transcript with significantly different levels between the MD and WT samples was *Nr2f1* (P<0.001). This gene is located adjacent to the gene desert at a genomic distance of approximately 800 Kb from the *Mcs1a* orthologous locus. To verify that *Nr2f1* is indeed a target gene, we used TaqMan gene expression assays on additional mammary gland samples. *Nr2f1* was found to be downregulated by more than 80% in the MD samples as compared with the WT samples (Figure 2D, P<0.001). We also checked *Nr2f1* transcript levels in three other tissues. In thymus and ovary, we found that *Nr2f1* transcript levels are greatly reduced (Figure S1, P<0.001), to similar levels as the mammary gland. In the liver, however, *Nr2f1* transcript levels were not significantly different between MD and WT samples (Figure S1, P = 0.27), suggesting that there is some tissue-specificity in this regulatory mechanism.

### The non-protein coding *Mcs1a* locus regulates mammary gland transcript levels of *Nr2f1*

**Figure 3:**
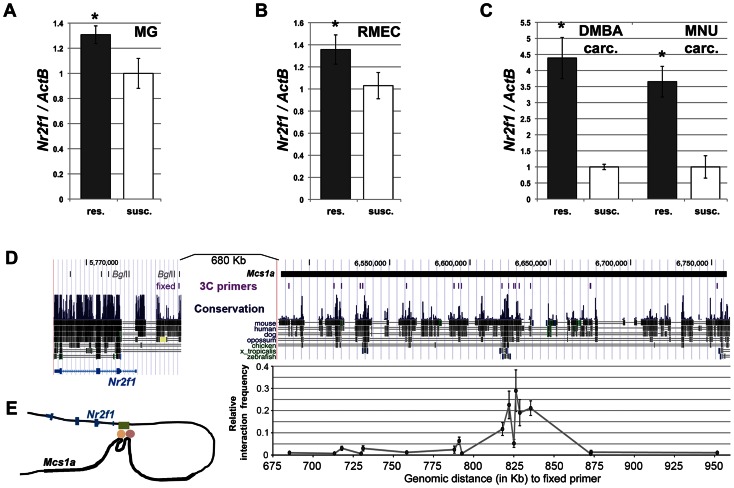
The non-protein coding *Mcs1a* resistance locus regulates transcript levels of *Nr2f1* in the mammary gland, mammary epithelium and mammary carcinomas. A–C) Q-PCR analysis of *Nr2f1* transcript levels in mammary gland (MG; panel A), rat mammary epithelial cell (RMEC, panel B) and DMBA- or MNU-induced mammary carcinoma (carc.; panel C) samples from resistant congenic (res.; n = 54 panel A, n = 18 panel B, n = 12 each panel C) and WF.Cop susceptible congenic control (susc.; n = 19 panel A, n = 11 panel B, n = 6 each panel C) rat lines. Data derived from both the W4 and W5 congenic lines are used in the *Mcs1a* resistant congenic data points. *Nr2f1* transcript levels are shown relative to the transcript level of the *ActB* endogenous control gene. D) Chromosome conformation capture (3C) assay for the *Nr2f1* promoter and the *Mcs1a* critical interval. The region is shown as a UCSC Genome Browser view (version rn4 of rat genome) and the location of the *Mcs1a* critical interval in indicated as a horizontal black line. The evolutionary sequence conservation track is also shown. The locations of the 3C assay primers are shown as vertical purple lines. The fixed primer in the *Nr2f1* promoter is shown with respect to *Bgl*II restriction sites in the *Nr2f1* gene span. Graphed is the average relative interaction frequency (+/− sem) of the *Bgl*II fragment in the *Nr2f1* promoter containing the fixed primer with each of the *Bgl*II fragments in *Mcs1a* containing the 3C assay primers (n = 4 or more templates). Significantly increased relative interaction frequency is indicated with 1 asterisk for a background cut-off interaction frequency of 0.05 and 2 asterisks for a background cut-off interaction frequency of 0.1. The horizontal axis indicates the genomic distance (in Kb) from the 3C assay primers in *Mcs1a* to the fixed primer in the *Nr2f1* promoter. The main peak in the interaction profile coincides with blocks of strong evolutionary sequence conservation (to zebrafish and frog, *X. tropicalis*). Sequence variation within the interacting *Mcs1a* region is outlined in Figure S2. E) Schematic drawing of the higher-order chromatin interaction of *Mcs1a* with the *Nr2f1* promoter. The *Mcs1a* critical interval is indicated as a thick area in the black line that represents the DNA. The green, orange and red shapes represent the putative DNA-binding proteins involved in the structure.

Considering *Nr2f1* as the main target of the *Mcs1a* locus, we also looked at its transcript levels in mammary glands (MG), rat mammary epithelial cells (RMECs) and mammary carcinomas (carc.; induced by DMBA and MNU) from susceptible congenic control (WF.Cop) and *Mcs1a* resistant congenic rats. The resistance allele was provided by the W4 or W5 congenic lines. Since the W4 and W5 lines did not differ significantly, data from both lines was included. For the RMECs, only data for the W4 resistant congenic line was obtained. The *Mcs1a* resistance allele was associated with increased *Nr2f1* levels in mammary gland (P = 0.01; Figure 3A) and RMEC (P = 0.02; Figure 3B). Both DMBA- and MNU-induced carcinomas from *Mcs1a* resistant congenic animals had strongly increased *Nr2f1* transcript levels, as compared with DMBA- and MNU-induced carcinomas from susceptible control congenic rats (P<0.001; Figure 3C).

As the *Mcs1a* locus is located at a genomic distance of over 800 Kb from *Nr2f1*, we asked if a chromatin looping structure exists that would support such long distance regulation. The chromosome conformation capture (3C) assay was developed to detect higher-order chromatin interactions for any locus of interest [39], [40]. To apply this methodology and investigate higher-order chromatin interactions between *Mcs1a* and *Nr2f1*, RMECs were fixed using formaldehyde to crosslink proteins and DNA, thus capturing interacting chromatin fragments. Crosslinked chromatin was digested using the *Bgl*II restriction enzyme and ligated in a large volume (of 7 ml). The large volume ligation reaction reduces random ligations and favors ligations of crosslinking-captured DNA fragments. Ligation events were detected and quantified using PCR and agarose gel electrophoresis. The PCR detection assay was designed such that the fixed primer was located in the rat *Nr2f1* promoter (Figure 3D). The experimental primers were located within the *Mcs1a* critical interval (Table S2), overlapping with areas of the strongest evolutionary conservation (Figure 3D). Each experimental primer was tested in combination with the fixed primer on the 3C templates and a positive control (BAC-derived) template. The relative interaction frequency of two chromatin fragments represented by a primer pair equals the PCR signal intensity of the RMEC 3C template relative to that of the positive control template. We found an area of multiple *Bgl*II restriction fragments with increased relative interaction frequencies above background levels (P<0.05) to exist within the *Mcs1a* critical interval (Figure 3D). The main peak coincides with genetic elements of the highest evolutionary conservation present in *Mcs1a*. These findings suggest that a putative regulatory element within *Mcs1a* forms a higher-order chromatin structure with the *Nr2f1* promoter over 820 Kb of genomic sequence (Figure 3E). None of the interactions is significantly different between the susceptible and resistant *Mcs1a* genotypes, suggesting that the DNA-binding proteins facilitating the interactions do not involve polymorphic sites. To identify genetic variants within and in the vicinity of the looped fragments that may explain *Nr2f1* transcript regulation, we resequenced approximately 12.5 Kb of the genomic region involved in the higher-order chromatin structure in the WF and Cop parental inbred strains and found 17 genetic variants (Figure S2; Table S3). For only 1 variant, the resistance (Cop) allele (a 14 bp deletion) is predicted to disrupt a rat-mouse-human-conserved binding motif, namely COUP-TF (V$Coup_01; Figure S2). As the *Mcs1a* resistance allele is associated with increased *Nr2f1/Coup-tf1* expression (Figure S2), it is possible that the 14 bp deletion polymorphism of the *Mcs1a* resistance allele omits a *Nr2f1* self-repressive gene expression modulatory function that acts through the intact COUP-TF binding motif on the susceptible *Mcs1a* allele. Such regulatory function would have to be investigated in detail in the future.

### *Mcs1a* affects MEC proliferation and differentiation

**Figure 4:**
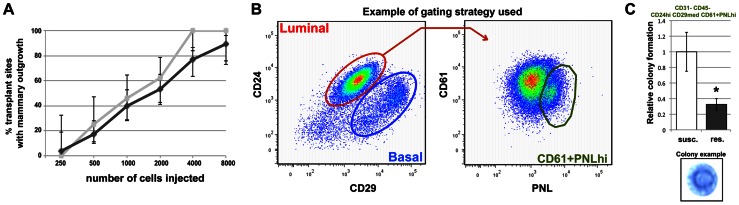
*Mcs1a* affects rat mammary epithelial cell (RMEC) growth and differentiation. A) Results of the limiting-dilution RMEC transplantation assay. Graphed is the percentage of transplant sites with an outgrowth versus the number of RMECs transplanted. Vertical bars represent lower and upper limits of a 95%-confidence interval for a proportion. Outgrowth potential of RMECs from the susceptible congenic control (susc.; light grey) and *Mcs1a* resistant congenic (res.; dark grey) line (lines W4 and W5 combined) is not significantly different. B) Pseudocolor dot plots from a susceptible congenic control sample, representing the gating strategy used to enrich for clonogenic luminal RMECs using cell sorting on the BD FACS Aria. CD31–CD45− RMECs were divided into luminal and basal cells based on CD24 and CD29 expression (left panel). Based on staining with anti-CD61 and peanut lectin (PNL), the clonogenic luminal cell population (CD45–CD31-CD24hiCD29medCD61+PNLhi; Figure S3) was selected for Matrigel assays (right panel). C) Graphed is the average (+/− sem) number of spherical mammary colonies formed in Matrigel assays plating 10,000 clonogenic RMECs sorted from the susceptible congenic control (n = 12) and *Mcs1a* resistant congenic line (n = 10; line W4 only). In the lower panel, a representative picture of a spherical mammary colony in a methylene blue-stained Matrigel is shown. Significantly different colony-forming ability (P<0.05) is indicated by an asterisk.

Since *Mcs1a* is mammary cell-autonomous, we asked if the locus has an effect on MEC biology, i.e. proliferation and differentiation. First, we tested for differential repopulating ability of RMECs from susceptible congenic control (WF.Cop) and *Mcs1a* resistant congenic animals. The resistance allele was provided by the W4 or W5 congenic lines. Differential repopulating ability could be indicative of a quantitative or functional difference in the mammary stem cell pool potentially underlying the susceptibility phenotype. Freshly isolated RMECs from *Mcs1a* resistant congenic animals and susceptible congenic control animals were grafted into the interscapular white fat pads of recipient animals of the same genotype. A dilution series of 250, 500, 1000, 2000, 4000 and 8000 cells was tested (Figure 4A). Six weeks after transplantation, the interscapular fat pads were harvested and scored for presence of mammary ductal structures as previously described [41]. The repopulating ability (determined by the estimated number of cells required to give 50% outgrowth occurrence) of RMECs from the susceptible congenic control animals was found not to be different (P>0.05) than that of the *Mcs1a* resistant congenic animals of either line W4 or W5. Another statistical approach was taken to seek for a possible difference in outgrowth potential at each cell number individually between the susceptible and resistant (W4 and W5 combined) genotypes. Therefore, Chi-square tests for independent distributions in a 2×2 contingency matrix were conducted. At all cell numbers the outgrowth potential for the resistant genotype was not different (P>0.05) than that of the susceptible genotype. These data suggest that the *Mcs1a* allele does not affect mammary stem cell activity.

In the next experiment, we tested the colony-forming ability in Matrigel of a purified population of clonogenic RMECs from *Mcs1a* resistant congenic (line W4) and susceptible congenic control (WF.Cop) animals. This assay tests the proliferating potential of the clonogenic RMEC pool. Freshly isolated single RMECs were antibody-stained and sorted using FACS. Gating strategies were used to exclude hematopoietic cells (CD45) and endothelial cells (CD31). From the RMEC-enriched (CD31–CD45−) population, the luminal cells (CD24hiCD29med) were selected and separated based on staining with anti-CD61 and peanut lectin (PNL; Figure 4B). We chose to focus on the luminal population, since CD61hi luminal cells have previously been identified in the mouse as the luminal progenitor population [22] and PNLhi clonogenic rat cells were previously found to overlap largely with the luminal population [25]. The colony-forming ability of 3 sorted cell fractions was tested, namely luminal CD61hiPNL+, CD61+PNLhi and CD61+PNL+ (Figure S3). From each sorted fraction, 10,000 cells were plated in Matrigel for each well. After 10 days of culturing, the colony-forming ability was determined by counting the spherical mammary colonies (Figure 4C, lower panel). For every sorted sample, we found that the CD61+PNLhi cell fraction had a ∼6-fold (P<0.001) increased colony-forming ability and the CD61hiPNL+ cell fraction a ∼1.6-fold (P = 0.02) decreased colony-forming ability, as compared with the CD61+PNL+ cell fraction (Figure S3), verifying that in rats PNLhi luminal MECs are enriched with progenitor cells. When comparing the susceptible congenic control and the *Mcs1a* resistant congenic samples, we found that the CD61+PNLhi cell fraction from the susceptible congenic control samples had a more than 2-fold increased colony-forming ability (Figure 4C; P = 0.02). These results indicate that the susceptible congenic control luminal RMEC fractions have increased proliferative capacity as compared with the *Mcs1a* resistant congenic RMEC fractions.

**Figure 5:**
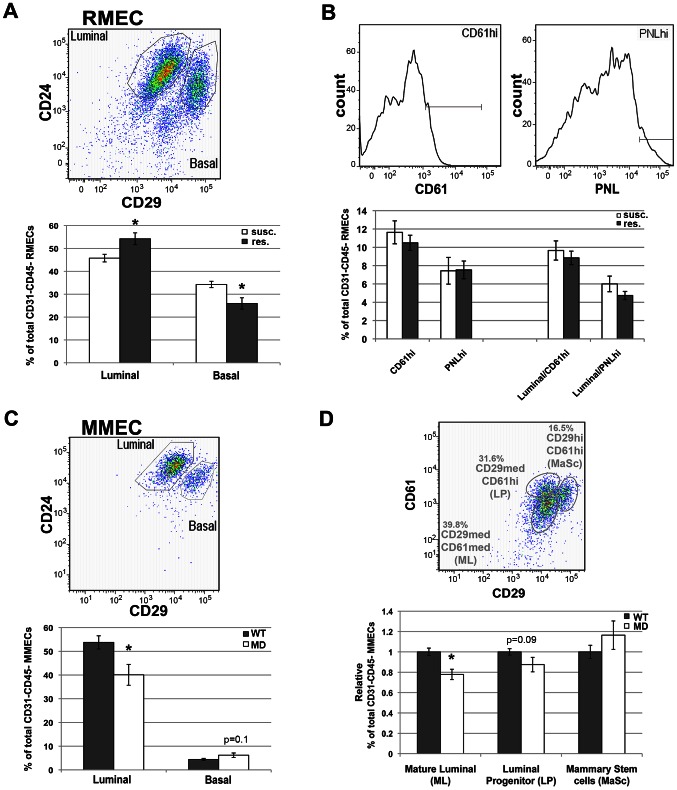
FACS analysis of rat and mouse MEC populations. A) Quantification of luminal and basal RMEC populations derived from susceptible congenic control (susc.; open bars; n = 6; WF.Cop) and *Mcs1a* resistant congenic (res.; filled bars; n = 7; Line W4) rats. B) Quantification of the CD61hi and PNLhi gates in RMEC populations derived from susc. (open bars; n = 11; WF.Cop) and res. (filled bars; n = 24; Lines W4 and W5). These gates were quantified in all CD31–CD45− RMECs as well as in the luminal RMECs. PNL = peanut lectin. C) Quantification of luminal and basal MMEC populations derived from wild type (WT; filled bars; n = 30) and megadeletion (MD; open bars; n = 27) mice (FVB and B6 pooled). D) Quantification of mature luminal (ML), luminal progenitor (LP) and mammary stem cell (MaSc) populations derived from WT (filled bars; n = 30) and MD (open bars; n = 27) mice (FVB and B6 pooled). FACS pseudocolor dot plot or histograms in each panel's upper figure illustrate the gating strategies used to quantify specific MEC populations. These dot plots were taken from a susc. sample (RMEC), or from a WT (FVB) sample (MMEC). Graphed in panels A–C are the average (+/−sem) percentages of populations among CD45–CD31− MECs. Graphed in panel D is the average (+/− sem) percentage of population among CD45–CD31− MECs, expressed relative to the WT run on the same day. In all graphs, significantly different (P<0.05) percentages of cells between susc. and *Mcs1a* or between WT and MD are indicated with an asterisk.

We also looked for quantitative differences in RMEC differentiation between susceptible congenic control (WF.Cop) and the *Mcs1a* resistant congenic animals. The resistance allele was provided by the W4 or W5 congenic lines. Fresh RMECs were obtained and stained for FACS analysis, as described previously [25]. The *Mcs1a* resistant congenic animals have more luminal (P = 0.02) and less basal (P = 0.02) RMECs as compared with susceptible congenic animals (Figure 5A), which significantly shifts the luminal-to-basal ratio by 34% (P = 0.008). The abundance of PNLhi or CD61hi subpopulations (Figure 5B) as well as the mean fluorescence intensities (not shown) of these stainings were not different between susceptible and resistant *Mcs1a* congenic animals. In addition, we looked at MMEC differentiation in WT and MD mice by FACS analysis, as described previously [42]. Like for the rat, we used antibodies against CD45 and CD31 to exclude hematopoietic and endothelial cells, respectively, and antibodies against CD24 and CD29 to quantify luminal and basal MMEC populations (Figure 5C). Like for *Mcs1a* congenic resistant and susceptible control rats, we found a shift of 36% in luminal-to-basal ratio between WT and MD mice. MD mice had a less abundant luminal and a trend towards a more abundant basal population (Figure 5C, P<0.001 and P = 0.10 for luminal and basal, respectively). Similar results were obtained for the MD mutation on both genetic backgrounds (FVB, B6). We also performed this analysis based on CD29 and CD61 expression to quantify mature luminal (ML, CD29medCD61−), luminal progenitors (LP, CD29medCD61+) and mammary stem/progenitor cells (MaSc, CD29hiCD61+). As compared with WT mice, the MD mice had a less abundant mature luminal population (P<0.001), a trend towards a less abundant luminal progenitor population (P = 0.09) and no significantly different mammary stem/progenitor cell population (P = 0.26; Figure 5D), suggesting that the *Mcs1a*-associated gene desert locus primarily affects luminal differentiation. Both MD mice and susceptible congenic control rats have a lower abundance of luminal cells and lower *Nr2f1* transcript levels, as compared with WT mice and *Mcs1a* resistant congenic rats, respectively, suggesting that *Nr2f1* modulates luminal MEC differentiation.

Next, we tested if short interfering RNA (siRNA)-mediated knockdown of *Nr2f1* transcript levels in cultured human breast cells is capable of directly influencing the cellular differentiation pattern. The human breast cancer cell line MCF7 and breast epithelial cell line MCF10A were transfected with siRNAs against *NR2F1* (siNR2F1) and non-targeting control siRNA (siCONTROL). No morphological differences were observed between cells transfected with siNR2F1 or siCONTROL. At 40 hours after transfection, cells were harvested for *Nr2f1* expression analysis and stained for FACS analysis with fluorescently labeled antibodies against commonly used markers of MEC differentiation, namely CD24, CD29, CD44 and CD49f. As expected, the siNR2F1-treated cells have an over 2-fold reduction of *NR2F1* transcript level, as compared with siCONTROL-treated cells (Figure S4). In both MCF7 and MCF10A we found that *NR2F1* knockdown upregulated CD24, as compared with treatment of the cells with non-targeting siRNAs. None of the other markers of differentiation was affected by *NR2F1* knockdown (Figure S4), suggesting that *Nr2f1* transcript levels have a direct effect on cellular differentiation through upregulation of CD24.

### Global gene expression analysis reveals a proliferative expression signature associated with low *Nr2f1* transcript levels in mouse mammary gland and human breast cancer

**Figure 6:**
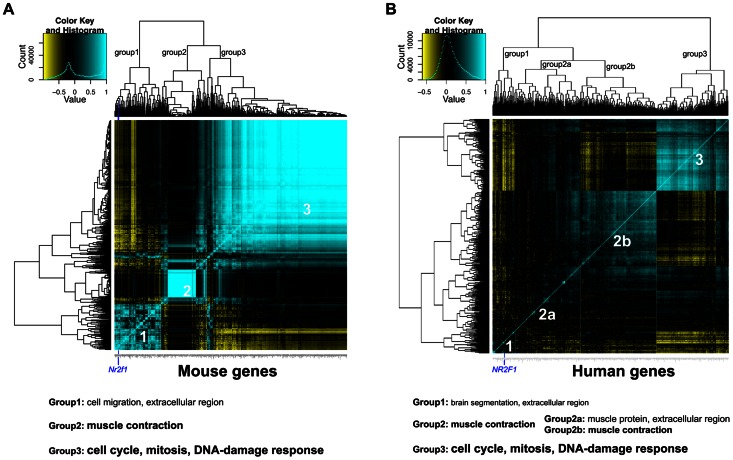
Global gene expression analysis reveals potential processes associated with *Nr2f1/NR2F1* transcript levels. A) Heatmap of expression correlation clustering analysis of 412 genes (which have 1-1-1 mouse-rat-human orthologues) that are differentially expressed between mammary gland samples from megadeletion (MD) and wild type mice (WT), both FVB. B) Heatmap of expression correlation clustering analysis of the same 412 genes in 243 human breast cancers from GSE3494, downloaded from the Gene Expression Omnibus. For both panels, strong correlation is indicated in blue, strong anti-correlation is indicated in yellow. The position of *Nr2f1/NR2F1* in group 1 is indicated by a dark blue vertical line. Below the panels, a summary of the gene ontology enrichment analysis is given. Smaller print indicates weak enrichment, larger print indicates strong enrichment (Tables S5, S6).

In the global RNA-seq expression analysis, 1,531 genes were found to be differentially expressed (DE) between the mammary gland samples from MD and WT (FVB) mice (Table S4). Of these, 412 genes have annotated 1-1-1 mouse-rat-human orthologues. *Nr2f1* is listed in the top 10 genes with the lowest P-value and is the top of the list of genes with 1-1-1 mouse-rat-human orthologues (Table S4). We applied a gene expression correlation clustering analysis using the 412 DE genes with 1-1-1 orthologues. The DE genes mainly clustered into three groups and *Nr2f1* is found in the first group (Figure 6A). To functionally annotate the groups of correlated genes, two online gene ontology (GO) category enrichment calculation tools were used, namely the Gene Ontology enRIchment anaLysis and visuaLizAtion tool (GOrilla) and the Database for Annotation Visualization and Integrated Discovery (DAVID) [43], [44]. The *Nr2f1* containing group is weakly enriched for genes related to cell migration, the extracellular matrix and innate immunity/inflammation (Table S5). The second group of strongly correlated genes was found not to correlate or anti-correlate with groups 1 and 3. This group was enriched for genes related muscle contractile function (Table S5). Group 3 is anti-correlated to the *Nr2f1-*containing group and is strongly enriched in genes related to the cell cycle, proliferation and DNA-damage response (Table S5). These results implicate that reduced *Nr2f1* transcript levels in the MD mammary gland is associated with an increased expression of cell cycle-related genes, which may render the mammary gland in a more proliferative state.

From the publicly available breast cancer global gene expression study GSE3494 containing data for 243 breast cancers [45], we selected 412 human probe sets from the Affy U133a array that are annotated to correspond to the 412 mouse DE genes. By performing the same correlation clustering analysis, an expression correlation pattern was identified to consist of 3 groups (Figure 6B). Group 1, again, is the *NR2F1*-containing group, weakly enriched with genes involved in developmental processes such as brain segmentation and morphogenesis (Table S6). Group 2 is now a large group that can be split up into subgroup 2a and 2b. Group 2 as a whole and subgroup 2b are strongly enriched with muscle contraction-related genes, whereas subgroup 2a is weakly enriched for muscle protein- and extracellular-related genes and is considered to be a mix between group1 and 2. Similar to the mouse mammary gland correlation analysis, group 3 is very strongly enriched for cell cycle/proliferation and DNA-damage response-related genes and is anti-correlated to the *NR2F1*-containing cluster (Table S6), suggesting that also in human breast cancer low *NR2F1* transcript levels are associated with an increase in cell cycle-related gene expression.

Next, we looked if the similarities in gene expression patterns between the mouse MD mammary gland and the human breast cancer data set hold up within the genome-wide data sets. From the GSE3494 global gene expression study, we selected 9,828 human probe sets from the Affy U133a array that are annotated to have 1-1-1 mouse-rat-human orthologues. First, we checked for similarities between the human and mouse data sets in gene lists anti-correlated to *Nr2f1* transcript levels. From the list of 126 human genes in the clustered group of genes anti-correlated to *NR2F1* transcript levels (Group 3, Table S6), 51 and 64 genes were found to be present in the top 100 and 200 anti-correlated genes to the *Nr2f1* transcript level in the mouse study, respectively. The probability that 51 or 64 of the anti-correlated genes (Group 3) would be in the top 100 or 200 anti-correlated from all 9,828 genes by chance would be <10^−74^ or <10^−78^, respectively, suggesting high similarity in genes anti-correlated to *NR2F1/Nr2f1* between human breast cancer and the mouse MD mammary gland. Similar analysis for the 37 genes in the *NR2F1*-containing cluster (Group 1, Table S6) yielded 3 and 5 genes in the top 100 and 200 genes correlated to the *Nr2f1* transcript level, which translates to a probability of this occurring randomly of 0.000571 and 0.000106, much higher than the probabilities for the anti-correlated genes.

We also functionally explored the genes most correlated and anti-correlated to *NR2F1/Nr2f1* in both the human and mouse data set, regardless of the occurrence of the genes in the mouse DE gene-based cluster analysis. From the 59 genes with strongest anti-correlation (r<−0.3) to *NR2F1* in the human breast cancer data set, 41 (69%) were also found to be among the strongest anti-correlated (r<−0.3) to *Nr2f1* in the mouse MG data set, which is 2-fold enrichment in comparison to strongly anti-correlated genes (r<−0.3) to *Nr2f1* in the entire mouse data set (34%). These 41 genes are found to be strongly enriched with cell cycle/proliferation-related genes (Table S7). From the 297 genes with strongest correlation (r>0.3) to *NR2F1* in the human breast cancer data set, 64 (22%) were also found to be the strongest correlated (r>0.3) to *Nr2f1* in the mouse MG data set, which is 1.3-fold enrichment in comparison to equally strongly correlated genes (r>0.3) to *Nr2f1* in the entire mouse data set (17%). These 64 genes are found to be enriched with genes involved in a wide variety of processes, including extracellular matrix and developmental processes, as well as signaling pathways (Table S7). These analysis suggest that the human breast cancer and the MD mouse MG gene expression data sets are particularly similar in genes anti-correlated with *NR2F1/Nr2f1*, which are strongly enriched with genes involved in cell cycle/proliferation.

Proliferation gene signatures have been explored for usage as prognostic markers in breast tumor expression studies [46]. Upregulation of such genes in breast cancer is generally indicative of poor prognosis [47], [48]. In a study to identify a novel gene list for “breast cancer intrinsic” subtype classification, a 20-gene proliferation signature was found to form one of the predictive modules [49]. Of these 20 genes, we found 14 to have human-rat-mouse orthologues of which 10 were DE in the mouse MG RNA-seq study with all 10 genes upregulated in the MD samples. The probability of selecting by chance 10 of these 14 proliferation signature genes into the 412 mouse DE gene set out of a total of 9,828 genes is lower than 10^−11^. This result suggests that the MD mammary gland gene expression profile (with low *Nr2f1* transcript levels) shows signs of a proliferative environment. This result is in accordance with the result from the Matrigel assay that indicated an increased colony-forming ability for selected cells from the susceptible mammary gland (with lower *Nr2f1* transcript levels) as compared to those from the resistant congenic mammary gland.

### *NR2F1* transcript levels in human breast cancer

**Figure 7:**
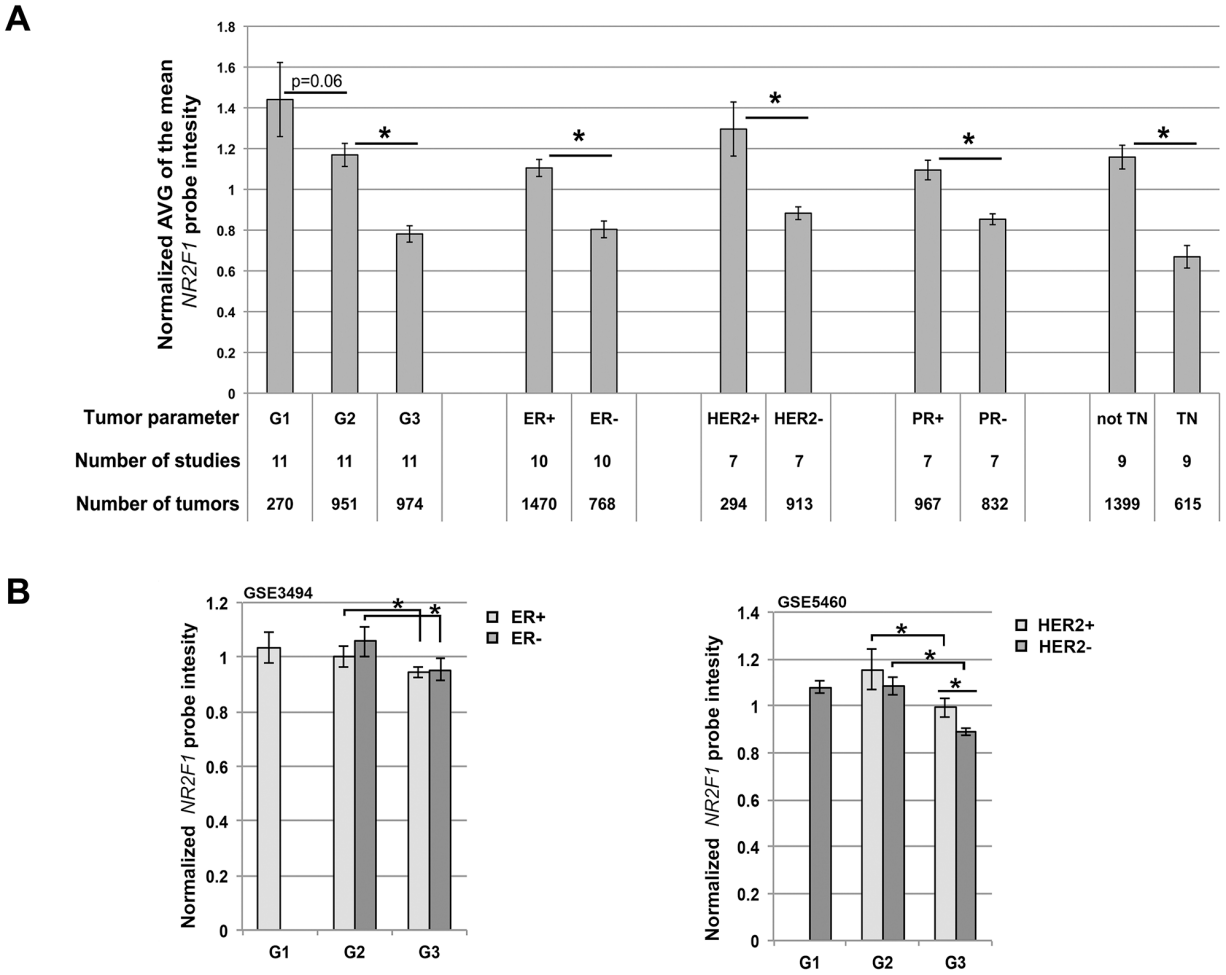
Reduced *NR2F1* transcript levels in human breast cancer correlate with histological grade 3 tumors. A) Average (+/− sem) of the normalized median *NR2F1* probe intensities, obtained from a total of 12 breast cancer expression studies (with 120+ samples each study) available through Oncomine. G = Histological grade, ER = Estrogen receptor, PR = Progestrone receptor, HER2 = Human epidermal growth factor receptor 2, TN = Triple-negative. B) Average (+/− sem) of the normalized *NR2F1* probe intensity in sample groups organized by grade and ER status (from GSE3494; left panel) or by grade and HER2 status (from GSE5460; right panel). Left panel: ER+G1 n = 62, ER+G2 n = 116, ER+G3 n = 33, ER-G2 n = 11, ER-G3 n = 21. Right panel: HER2+G2 n = 7, HER2+G3 n = 23, HER2-G1 n = 27, HER2-G2 n = 24, HER2-G3 n = 46. Significantly different *NR2F1* transcript level (P<0.05) is indicated by an asterisk.

Since *Nr2f1* is implicated in MEC proliferation and differentiation in mice and rats, we asked if *NR2F1* transcript levels correlate with clinical features of human breast cancer. The Oncomine database encompasses a comprehensive listing of breast cancer gene expression studies, including available clinical information on the samples [50]. When including 12 studies with 120+ samples for each study we found that the average of the median *NR2F1* transcript levels reduces with increased histological grade (Figure 7A). Histological grade 3 tumors are more proliferative and more poorly differentiated than grade 1 or 2 tumors. The therapy-resistant and most aggressive form of breast cancer, based on hormone receptor status is the ‘triple-negative’ class of tumors that are more likely to be of grade 3 when resected. Median *NR2F1* transcript levels were found to be lower in triple-negative breast cancers, as compared with ‘receptor positive’ (non-triple-negative) breast cancers (Figure 7A). In accordance with this finding, estrogen receptor (ER)-negative and progesterone receptor (PR)-negative breast cancers had lower median transcript levels of *NR2F1* as compared with their positive counterparts (Figure 7A). Notably, *NR2F1* transcript levels were found to be lower in human epidermal growth factor receptor 2 (HER2)-negative breast cancers (that mostly are ER-positive), as compared with the more aggressive HER2-positive breast cancers (Figure 7B).

Finally, we asked if *Nr2f1* transcript level anti-correlated with histological grade within both ER-positive/ER-negative and within both HER2-positive/HER2-negative breast cancer subtypes. For 2 of the 12 previously mentioned breast cancer gene expression studies we obtained the raw data from the gene expression omnibus (GEO; GSE3494 and GSE5460). The GSE3494 data set consists of microarray gene expression data for ER-positive and ER-negative breast tumors including histological grade 2 and 3 tumors in both ER classes and the GSE5460 data set consists of data for HER2-positive and HER2-negative breast tumors including histological grade 2 and 3 tumors in both HER2 classes. We found that *NR2F1* transcript levels are significantly lower in grade 3 tumors as compared with grade 2 tumors in both ER classes (Figure 7B, left panel), as well as in both HER2 classes (Figure 7B, right panel). Additionally, in the GSE5460 data set the grade 3 HER2-positive breast cancers were found to have higher *NR2F1* transcript levels as compared with the grade 3 HER2-negative breast cancers (Figure 7B, right panel), suggesting a regulatory effect of *HER2* amplification on *NR2F1* transcript levels. In summary, these analyses indicate that low transcript levels of *NR2F1* are strongly associated with high histological grade, poorly differentiated, highly proliferative breast cancers, including therapy-resistant ‘triple-negative’ breast cancer.

## Discussion

The inherited portion of breast cancer susceptibility is complex and likely involves numerous genetic factors [51], [52]. With the results from genome-wide association studies (GWAS) it became clear that the genetic landscape of breast cancer susceptibility largely consists of low-penetrance alleles that are common in the population [53]. Many such alleles are located in non-protein coding regions of the genome, including in gene deserts, such as the one on human chromosomal band *8q24* [5]. Mechanisms underlying the genetic associations are largely unknown. It is generally hypothesized that non-protein coding variants could modulate disease processes through the regulation of gene expression.

Like for human breast cancer susceptibility, many loci associated with rat mammary cancer susceptibility have been discovered over the last decade [54]. Multiple of these QTL have been identified by our laboratory through linkage analysis and fine-mapping using congenic recombinant lines [32], [33], [55], [56], [57]. For some of the rat loci, common alleles associated with breast cancer susceptibility in the human orthologous loci were found [58], [59]. One major advantage of such rat-human comparative genetics approach is the availability of a highly relevant genetic mammalian model system for mechanistic studies [30]. Understanding how loci affect breast cancer susceptibility will be informative in the design of preventative or early intervention strategies that would be applicable to many women at risk.

In this study, we identified a 277 Kb critical interval for the previously discovered *Mcs1a* resistance allele that is derived from the Cop inbred rat strain [33]. The allele when introgressed on the susceptible genetic background from the WF inbred rat strain modulates mammary carcinoma multiplicity by approximately 50%. The protective effect of the allele works against three distinctly acting carcinogenic treatments, indicating that the mechanism modulates mammary carcinogenesis beyond a carcinogen-specific initiation stage. Since the susceptibility or resistance phenotype of the WF or Cop *Mcs1a* allele, respectively, is transferrable in a mammary gland transplantation/carcinogenesis study, we concluded that the locus modifies mammary carcinoma development in a mammary cell-autonomous manner. This is in contrast to the Wistar-Kyoto (WKy) inbred strain-derived *Mcs5a* resistance locus, for which we previously published a non-mammary cell-autonomous mode of mammary carcinoma development modulation using a similar transplantation/carcinogenesis assay [60].

Markedly, the location of the 277 Kb critical interval lays within a 3 Mb gene desert, which classifies the *Mcs1a* allele as non-protein coding. To identify the gene targets for regulation by the *Mcs1a* associated non-protein coding region, we developed a mouse model lacking a 535 Kb gene desert region orthologous to *Mcs1a*. The mouse model organism was chosen, as a large deletion (i.e. megadeletion; MD) engineering resource by means of MICER vectors was readily available. A MICER vector-assisted large deletion mouse model had aided before in discovering the gene targets of regulation by a non-protein coding region associated with coronary artery disease [61]. At the time we developed our MD mouse model, targeted rat genetic manipulation technologies were still under development. With the current maturation of zinc-finger nuclease-mediated genome editing technology [62], [63], a similar MD approach will be applicable to the rat model organism in the near future. Using RNA-seq we characterized mammary gland gene expression of MD and WT mice. Of genes surrounding the gene desert within 2.5 Mb, we only found the transcript level of the orphan nuclear receptor gene *Nr2f1/Coup-tf1* to be strongly reduced upon deletion of the non-protein coding region. In addition, in the global RNA-seq gene expression analysis, *Nr2f1* had the lowest P-value of all differentially expressed genes with annotated 1-1-1 mouse-rat-human orthologs. This gene is located at a genomic distance of over 800 Kb from the MD mutation, suggesting the presence of a strong *Nr2f1* distal enhancer in the deleted region. *Nr2f1* transcript levels were found to be downregulated in whole mammary gland, RMEC and mammary carcinoma samples from susceptible congenic controls as compared with *Mcs1a* resistant congenic rats. These results identify *Nr2f1* as a strong candidate breast cancer susceptibility gene whose increased mammary transcript levels are associated with resistance to mammary carcinoma development. It is worth mentioning that the difference in *Nr2f1* transcript levels between the susceptible and resistant *Mcs1a* alleles are more substantial in tumors as compared with untransformed cell types of the mammary gland. A plausible explanation for this observation could be that *Nr2f1* transcript levels in an unidentified progenitor (or perhaps cancer-initiating) RMEC population may show similar substantial differences, which could be masked by other cell types present in the whole mammary gland or RMEC samples. The presence of a higher-order chromatin structure connecting the *Nr2f1* promoter with a strongly conserved element within *Mcs1a* supports the long-range (∼820 Kb) regulatory potential of the *Mcs1a* locus. It should also be noted that the 3C assay was biased towards elements with the strongest evolutionary conservation (through fishes). Potentially interesting interacting elements in less conserved sequences may have been overlooked. Because the intensity of the chromatin loop is not affected by the *Mcs1a* alleles, but *Nr2f1* transcript levels are, we hypothesize that the proteins involved in the higher-order chromatin interaction may be not be the same factors regulating *Nr2f1*. Resequencing of the interacting region in the Cop and WF parental inbred strains revealed the presence of 17 polymorphisms. Only one polymorphism, a 14 bp deletion in the Cop strain, affects a human-rat-mouse conserved binding motif, which is a COUP-TF binding site. Since the resistance (Cop) allele is associated with increased *Nr2f1* (*Coup-tf1*) transcript levels we hypothesize that the 14 bp deletion removes a repressive autoregulatory module of *Nr2f1* communicating with its own promoter. Since the MD mutation of the entire locus profoundly downregulates *Nr2f1* transcript levels, the entire region is acting as a strong enhancer. Thus, we propose that in the susceptible strain harboring the WF allele with the intact COUP-TF binding motif, the repressive autoregulatory mechanism may modulate *Nr2f1* transcription in the context of the activity of the enhancer. A germ-line mutation in the orthologous binding motif is not known to exist in mice or humans. Other variants outside of the conserved element (perhaps located in closer genomic proximity to the *NR2F1* promoter) may confer similar *NR2F1* regulation and thus potentially associate with breast cancer risk.

By taking advantage of the available congenic rat and genetic mouse models, we focused on dissecting the mechanisms underlying the non-protein coding *Mcs1a* locus on the organismal level. Because of the mammary cell-autonomous mechanism and change in mammary *Nr2f1* transcript levels, we looked for differences in MEC biology between susceptible and resistant *Mcs1a* congenic rats. We found in a limiting dilution RMEC transplantation assay for repopulation ability that mammary stem cell activity is not affected by *Mcs1a*. Next, we tested the proliferation potential of a specific clonogenic population of RMECs. Clonogenic RMECs have previously been described to stain brightly with peanut lectin (PNL) and to be mostly located within the luminal population [25], [26]. In this study, we show that the luminal clonogenic RMEC population with colony-forming ability in Matrigel is indeed marked by bright PNL staining and not by high CD61 expression, which was shown to enrich for luminal progenitor cells with colony-forming ability in the mouse mammary gland [22]. In later studies, c-kit and ALDH have been identified as more specific markers for luminal progenitor cell populations, illustrating the heterogenity of the luminal MEC population [23], [24]. In the future, these markers can be tested on RMECs in combination with PNL staining to pinpoint the rat luminal progenitor population, provided good antibodies are available for the rat. Interestingly, the colony-forming ability of the luminal PNLhi population was found to be reduced in animals carrying the *Mcs1a* resistance allele, as compared with susceptible controls. As determined by multiparameter FACS analysis of freshly isolated RMECs, the abundance of PNLhi and luminal PNLhi cells among CD31–CD45− RMECs was not significantly different. Hence, we concluded that the proliferation potential of the colony-forming luminal population is affected by *Mcs1a*. The FACS analysis, however, did reveal a RMEC differentiation phenotype associated with *Mcs1a*. Resistant congenic animals have a higher abundance of luminal and lower abundance of basal RMECs, as compared with susceptible congenic control animals. Basal RMEC are mainly characterized by high β1-integrin (CD29) expression and loss of β1-integrin in the mouse mammary gland impairs mammary cancer development [64], suggesting that lower abundance of basal RMECs in the resistant *Mcs1a* congenics may contribute to lower mammary carcinoma susceptibility. Interestingly, in both the *Mcs1a* congenic rat model and genetically engineered mouse model, higher expression of *Nr2f1* in the mammary gland is associated with higher abundance of luminal MMECs, identifying *Nr2f1* as a candidate MEC differentiation gene modulating luminal cell fate. Since in the MD mouse model the abundance of mature luminal cells was significantly affected, and the abundance of luminal progenitors and basal cells were not, we propose that the *Mcs1a*-associated locus may impact luminal cell fate through activities in the luminal progenitors. Several other genes have been shown to regulate luminal cell fate mainly through activities in luminal progenitors. Downregulation of *Gata-3* and upregulation of *FoxM1* have been demonstrated to lead to impaired luminal cell differentiation [22], [65]. Interestingly, we found the transcript level of *FoxM1* significantly upregulated in the MD mammary gland samples, whereas the *Gata3* transcript level was not affected (Table S4). *FoxM1* is predicted not to have a COUP-TF binding motif in its vicinity, thus the exact relationship of *FoxM1* and *Nr2f1* transcript levels remains to be investigated.

NR2F1 is an orphan nuclear receptor of the steroid hormone receptor superfamily [66]. Homodimers of NR2F1 bind the DR1 (direct repeats with 1 spacer) motif with the highest affinity [67]. NR2F1 is thought to act as a transcriptional repressor [68], [69], but can activate target genes as well [70], [71]. Nr2f1 has been previously recognized as an important factor in the development of the mouse nervous system [72], [73], [74] and the inner ear [75], [76]. Interestingly, ectopic *Nr2f1* expression in the developing telencephalon and knockdown of *Nr2f1* in primary neurospheres have been shown to result in defect neuronal cell fate determination [77], [78], suggesting that *Nr2f1* may function as a neuronal as well as a MEC differentiation gene. The MD mice generated in this study have normal bodyweight, lifespan, and startle response to finger flicking above the cage (to test for hearing loss), but do display a delayed eyelid opening phenotype. Delayed eyelid opening could be indicative of an eye development defect or an eyelid epidermal apoptotic defect [79]. Interestingly, Nr2f1 has previously been implicated in eye development and was found to be highly expressed in progenitor cells of the developing eye [80], suggesting that the delayed eyelid opening phenotype in the MD mice is likely due to aberrant *Nr2f1* expression in the differentiating eye progenitors.

There is a moderate amount of evidence that NR2F1 is involved in breast cancer, mainly through its cross-talk activities with the ER- [81], the arylhydrocarbon receptor- [82], and/or retinoic acid-mediated signaling [83]. Again, several studies emphasize NR2F1's dual role as a transcriptional repressor and activator, depending on the promoter its acting on and the cellular context, i.e. presence of other nuclear factors such as ER [84], [85]. We show in this paper that in all large human breast cancer gene expression studies examined, triple-negative (aggressive, therapy-resistant) and histological grade 3 (poorly differentiated, highly proliferative) breast cancers display lower *NR2F1* transcript levels as compared with ‘receptor-positive’ and histological grade 1/2 breast cancers, respectively. This observation is in accordance with the mouse and rat MEC differentiation and mouse mammary gland gene expression studies that show reduced *Nr2f1* transcript levels associated with less luminal differentiation and a more proliferative epithelial environment. Recently, *NR2F1* was presented in a breast cancer dormancy gene signature as a gene upregulated in dormant cells [86]. Notably, in the same study, MCF7 cells with siRNA-mediated depletion of *NR2F1*, when injected into the mammary fat pad of immunosuppressed mice resulted in increased growth as compared with negative control siRNA-treated MCF7 cells [86]. Again, consistent with our findings, this result provides functional evidence that lower *NR2F1* transcript levels increase the proliferative potential of breast cancer cells in an *in vivo* model system. In addition, we found in the MCF10A and MCF7 cell lines that siRNA-mediated reduction of *NR2F1* transcript levels results in increased expression of CD24. CD24 has previously been implicated in breast cancer, for example as a marker for the breast cancer-initiating cell population [87]. Ectopic expression of CD24 in breast cancer cells has been shown to result in increased proliferation, as well as cell motility and invasion [88] and the expression of CD24 in breast carcinomas has been associated with poor prognosis [89].

It should be noted that according to available HER2 status classification in the human breast cancer gene expression studies, the more aggressive HER2-positive breast cancers (also associated with poorer clinical outcome) were found to express higher *NR2F1* transcript levels, as compared with HER2-negative breast cancers (that are mostly ER-positive and less aggressive). In addition, we show for the GSE5460 gene expression data set that within both HER2 classes, histological grade 3 tumors have lower *NR2F1* transcript levels as compared with grade 2 tumors. In this data set, the grade 3 tumors from the HER2-positive class have higher *NR2F1* transcript levels as compared with grade 3 tumors from the HER2-negative class. We speculate that over expression of HER2 and subsequent stimulation of downstream signaling pathways increases *NR2F1* transcript levels. The cellular effects of higher *NR2F1* transcript levels may be very different for the amplified and unamplified HER2 backgrounds.

*NR2F1* is located on human chromosomal band *5q15*. Interestingly, a hotspot for copy number alterations (CNA) in breast cancer maps to chromosomal arm *5q* [90], [91], with deletions most frequently occurring at *5q11-5q34* [92]. These CNA have been associated with high histological grade, basal-like tumors, p53-mutation status, triple-negative tumors, and tumors from *BRCA1* carriers [90], [91], [93]. A recently published study describing comprehensive molecular portraits of human breast tumors identified the *5q* deletion hotspot as a large trans-eQTL, as the expression levels of hundreds of genes across the genome are associated with occurrence of *5q* deletions [93]. Interestingly, this study found the associated genes to enrich in GO categories involved in cell cycle processes, *FoxM1* transcriptional regulation and proliferation, many of which are also found in our MD and WT RNA-seq study to be anti-correlated to *Nr2f1* transcript levels. Placement of the *Mcs1a*-orthologous gene desert and *NR2F1* within the deletion hotspot suggests that *NR2F1* may play a role in deregulation of a fraction of these cell cycle-related genes associated with the triple-negative breast cancer-specific *5q* deletions.

**Figure 8:**
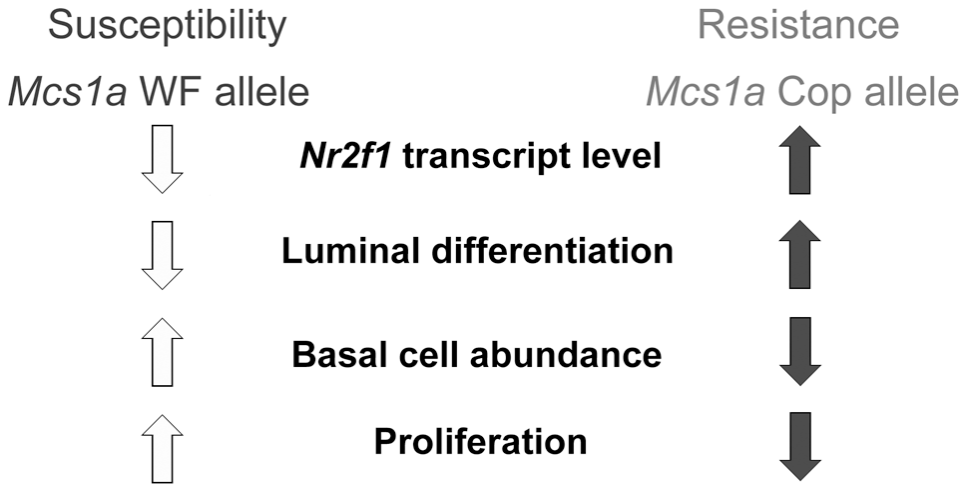
Proposed model for the *Mcs1a* breast cancer risk-affecting mechanism. The *Mcs1a* resistance allele displays increased mammary *Nr2f1* transcript levels as compared with the susceptible allele. Lower *Nr2f1* transcript levels in the mammary gland are associated with susceptibility, a lower percentage of luminal rat mammary epithelial cells (RMEC), a higher percentage of basal RMECs and increased colony-forming ability of the clonogenic luminal RMEC population, indicative of increased proliferation.

In summary, we describe the genetic dissection of the gene desert breast cancer susceptibility locus *Mcs1a*. We hypothesize that the resistance allele (from the Cop strain) carrying a truncated, potential transcriptionally suppressive COUP-TF (autoregulatory) binding motif, leads to increased *Nr2f1* transcript levels in the mammary gland, which increases luminal RMEC differentiation and creates a less proliferative, more differentiated mammary epithelium with decreased mammary carcinoma susceptibility (Figure 8). We present *NR2F1* as a strong candidate breast cancer susceptibility gene and MEC differentiation gene. In addition to a potential role in breast cancer susceptibility, we propose that reduced *NR2F1* transcript levels associated with the human breast cancer *5q* chromosomal deletions play a role in high-grade, poorly differentiated, proliferative breast cancer, including therapy-resistant triple-negative breast cancer. The human non-coding region orthologous to *Mcs1a* as well as *NR2F1* are located on chromosomal band *5q* in the region of frequent deletion. Raising *NR2F1* transcript levels, or enhancing NR2F1's activities has great potential as a strategy to aid in breast cancer prevention or breast cancer intervention, including for triple-negative breast cancer (and possibly excluding HER2-positive breast cancer). As a steroid hormone receptor family member, NR2F1 potentially is an attractive therapeutic target. To our current knowledge NR2F1 is still an orphan nuclear receptor, which means a ligand has not been identified yet. Based on strong amino-acid conservation of the NR2F1 ligand binding domain (96%) with that of NR2F2, the crystal structure of the NR2F2 ligand binding domain suggests that NR2F1 may also be activated by retinoic acid through coactivator recruitment-based release of its autorepressed conformation [94]. Identification of ligand-mediated activator mechanisms for NR2F1 is important to begin to exploit its therapeutic potential in the near future.

## Materials and Methods

### Ethics statement

All animal protocols were approved by the University of Wisconsin School of Medicine and Public Health Animal Care and Use Committee.

### Rats

The congenic rat lines were established and maintained in an AAALAC-approved facility as previously published [57]. Congenics are defined as genetic lines developed on a Wistar-Furth (WF; susceptible) genetic background and carrying the selected Copenhagen (Cop; resistant) *Mcs1a* alleles in homozygous fashion [33]. Resistant congenic lines with decreased susceptibility phenotypes (Q, R3, V4, W4, Y4, W5) carry Cop alleles that define the *Mcs1a* locus critical interval. The susceptible congenic lines (P5, V5, R5, A4, Y3) are WF-homozygous at the newly defined *Mcs1a* locus. The susceptible congenic control line (WF.Cop) was derived from congenic line W5 and is WF-homozygous for all *Mcs1* loci. The primer sequences for the genetic markers polymorphic between the WF and Cop inbred parental stains that are used to define the congenic lines are listed in Table S1.

### Carcinogenesis

Female rats, 7–8 weeks of age, were either orally gavaged with 7,12-dimethylbenz(a)anthracene (DMBA) at 65 mg/kg of body weight, injected intraperitoneally with *N*-nitroso-*N*-methylurea (MNU) at 50 mg/kg of body weight, or subjected to mammary ductal infusion of replication-defective retrovirus expressing the activated *HER2/neu* oncogene (*HER2/neu*) at a concentration of 1×10^5^ Colony Forming Units (CFU)/ml [34]. To obtain multiplicities, mammary carcinomas >3×3 mm were counted at 15 weeks post-DMBA, 15 weeks post-MNU, and 7 weeks post-*HER2/neu* treatment. Multiplicity data were statistically analyzed using Mann-Whitney nonparametric tests.

### Mice

All mice are maintained in an AAALAC-approved facility. The megadeletion mice were produced in collaboration with the University of Wisconsin Biotechnology Center Transgenic Animal Facility (http://www.biotech.wisc.edu/facilities/transgenicanimal). MICER clones (MHPP256h04; MHPN5k06; Sanger Institute, UK) were obtained and the vectors were purified. To ensure proper directionality of the construct upon insertion, the genomic insert from vector MHPP256h04 was flipped by *Asc*I digestion and religation. The vectors were prepared by creating a gap in the genomic insert, as efficient targeting using MICER vectors relies on the embryonic stem (ES) cell's gap repair mechanism [95]. AB2.2 (*HPRT* deficient) ES cells from the 129/SvEv strain were transfected by electroporation in the presence of a linear gap-containing MICER vector. The first electroporation was done in the presence of the (flipped) gap-containing MHPP256h04 vector. Puromycin-resistant ES cell clones were expanded and checked for proper targeting by Southern blot analysis (Figure S1). Following karyotyping, karyotypically normal clones were selected and expanded for targeting with the second MICER vector. After electroporation in the presence of the gap-containing MHPN5k06 vector, neomycin-resistant clones were expanded and checked for proper targeting by Southern blot analysis. A karyotypically normal doubly targeted ES cell clone was expanded. To excise the 535 Kb region (megadeletion, MD) between the *lox*P sites present in both inserted vectors, a Cre-recombinase expressing vector (pTurbo-Cre) was introduced through electroporation. As proper Cre-*lox*P recombination restores the functional *HPRT* gene [95], hypoxanthine/aminopterin/thymidine (HAT) resistant clones were expanded and checked for recombination by PCR using 2 primer combinations spanning the deletion (mmMICERdel:TGTCTAGAGCTTGGGCTGCAG mmMICERdel:AGACAGAATGCTATGCAACCT and Del2F:CATGGACTAATTATGGACAGG Del2R:CTCCTTCATCACATCTCGAGC). Karyotypically normal ES cell clones were monodispersed and microinjected into C57Bl/6 blastocysts to produce chimeric founders. After germ line establishment, the MD mutation was introgressed onto the FVB/N and C57Bl/6 inbred genetic backgrounds for >10 generations. Deletion of the 535 Kb region was further verified by PCR using 4 primer combinations within the deleted sequence (mmdelNeg:TGGACTTGATGTGCTCCTTG mmdelNeg:TGCCATCAATGAGTTTGAGG, 2F:AAGTGAAAGATGCTGACATTTCC 2R:AAGACTGAATTCTTGCCACTCAC, 3F:GGGAGCCATTTATCACAGTCCTA 3R:GACCCTCACAAAAGCTGGTTTA, 4F:ACACATTTGGAGATGCAAACAG 4R:CACAAAAGTCACCTAAAAGGATCA). Examples of genotyping images for primers mmMICERdel and mmdelNeg are shown in Figure S1.

### Mammary gland transplantation

Females from the susceptible (S) inbred strain WF, the resistant (R) congenic line Y4, and WFxY4 or Y4×WF intercross (F1) were used. Donor mammary glands with lymph nodes (LN) excised (both abdominal and adjacent inguinal glands) from 30–35 day old females were finely minced over ice and divided into four equal volumes. One volume was transplanted onto the interscapular white fat pad of each 30–35 day old recipient (1 donor/4 recipients). Three weeks after transplantation, all recipients were treated with DMBA. At 15 weeks post-DMBA, interscapular fat pads were examined for carcinoma development. In addition, each fat pad was whole mounted and stained with aluminum carmine to verify mammary gland establishment. As only 15 out of 228 rats developed multiple carcinomas in the transplant sites, the mammary carcinoma incidence data were analyzed as a binary response by logistic regression. The four transplant groups (S:S, S:F1, R:F1, R:R) form a 2×2 factorial design with donor and recipient genotypes as the main effects. Standard logistic regression was applied to the binary response data with two main effects and an interaction term.

### Quantitative real-time PCR

Female rats (11 weeks of age) of the susceptible congenic line WF.Cop (susc.) and resistant congenic lines W4 and W5 (res.), or female MD and WT mice (9 weeks of age; FVB) were used as tissue donors. RNA was extracted from snap-frozen mammary gland and mammary carcinoma tissues, from fresh RMEC samples, or from siRNA-treated human cell line samples. To synthesize cDNA from 800 ng of TURBO-free DNaseI-treated total RNA, the reverse transcriptase Superscript II kit (Invitrogen) was used according to manufacturer's directions. Quantitative real-time PCR was used to quantify transcript levels. TaqMan quantitative PCR primers and probes were ordered as premade assays (ABI/Applied Biosystems): rat *Nr2f1* Rn01489978_m1 (FAM), mouse *Nr2f1* Mm01354342_m1 (FAM), human *NR2F1* Hs00818842_m1 (FAM), rat *ActB* Rn00667869_m1 (VIC, endogenous control), mouse *ActB* Mm00607939_s1 (VIC, endogenous control) and human *GAPDH* Hs03929097_g1(VIC, endogenous control). Reactions were run as described previously [96]. Quantities of transcripts were measured by comparison of C_t_ values with a standard curve calculated from serial dilutions made from reverse transcriptase reactions that contained 2 µg of total RNA. Sample measurements are an average of three or four replicates within 0.5 C_t_ value. Sample measurements were normalized by dividing the gene specific transcript quantity over the endogenous control quantity. For each sample, the ratio was scaled to the average ratio of the control group from the same experiment, which are the susceptible congenic control group (rat), the WT group (mouse) or the siCONTROL-treated group (human cell lines). Data were analyzed using Mann-Whitney nonparametric tests.

### RNA-seq

RNA was extracted from snap-frozen mammary gland tissue from MD and WT mice (FVB) using the MagMax-96 Total RNA isolation kit (Ambion) according to manufacturer's directions. RNA samples were checked for integrity using Agilent 2100 Bioanalyzer. Two RNA samples were pooled using 5 µg of each for 10 µg of total RNA per library preparation sample. Sample preparation and next-generation sequencing was done at the University of Wisconsin Biotechnology Center Gene Expression Center. Sample preparation was done using the Rev.D mRNA sample preparation kit (Illumina), according to the manufacturer's recommendations. Samples were run on the Illumina GAIIx. Reads that made the quality cut-off were aligned to the mouse Ensembl set of 82,508 transcripts (http://genome.ucsc.edu) using Bowtie (v0.12.3; http://bowtie-bio.sourceforge.net/index.shtml) with the following settings: -f –v 1 −3 0 –a –m 100. Transcript levels were estimated using the RSEM algorithm [37]. Differential expression between the MD and WT samples was determined using edgeR [38]. Correlation analysis of gene expression was done on the 1,531 DE mouse genes and 412 DE mouse genes with 1-1-1 mouse-rat-human Affy (U133a probe set) orthologues. The Pearson correlation between the mean-centered RSEM tau expression values was calculated and visualized in R using the gplots library. Similar correlation analysis was done on the 412 human orthologous genes for which microarray data (Affy U133a) was available from a human breast cancer gene expression study. For this analysis, the GSE3494 dataset was downloaded from the Gene Expression Omnibus (GEO; www.ncbi.nlm.nih.gov/geo). The raw data was normalized using the RMA approach in R [97]. The online functional annotation tools GOrilla (http://cbl-gorilla.cs.technion.ac.il) and DAVID (http://david.abcc.ncifcrf.gov) were used to find Gene Ontology (GO) categories and biological processes enriched with DE genes [43], [44]. For the 412 DE mouse-rat-human orthologs genes analysis, the full mouse-rat-human orthologues gene list (9,828 genes, Table S5, S6) was used as the background list.

### Chromosome Conformation Capture (3C) assay

Templates were prepared from isolated RMECs from 6 animals of the susceptible congenic control line WF.Cop and 6 animals from the *Mcs1a* resistant congenic line, as described previously [96]. The restriction enzyme of choice was *Bgl*II. The fixed primer was chosen to be located in the predicted promoter of the *Nr2f1* gene. The experimental primers were chosen to be located within the *Mcs1a* critical interval, biased towards regions of evolutionary sequence conservation (Figure 3d). Primer sequences are listed in Table S2. The relative interaction frequency for each experimental primer in combination with the fixed primer was determined for each sample as the average of 3 replicate measurements divided by the average of a positive control (BAC-derived) template run in the same PCR plate [96]. A non-parametric Mann-Whitney test was performed to test for differences between genotypes. Since no genotype differences were detected, data for the *Mcs1a* profile were taken from all samples. Next, non-parametric Krukal-Wallis tests were performed to test for increased (P<0.05) interaction frequency of a primer pair above the background levels. The interaction frequencies of peak fragments were tested against interaction frequencies below two background cut-offs, namely 0.05 and 0.1.

### Resequencing

Genomic DNA was isolated from spleens of inbred WF and Cop rats using phenol and chloroform extractions. A total of 10 ng of genomic DNA was used in each PCR reaction. Primers were designed using Primer3 software and are listed in Table S3. Successful PCR reactions as verified by agarose gel electrophoresis were diluted 6 times. Of this dilution, 1 µl was used in a BigDye sequencing reaction in a total volume of 15 µl, according to the vendor's (Applied Biosystems) specifications with the exception that we used 0.7 µl BigDye enzyme mix. Sequencing reactions were cleaned-up using the CleanSeq kit (Agencourt) and submitted for sequencing through the UW Biotechnology DNA Sequencing Facility. Reads were visualized using 4Peaks software (Meckentosj) and scanned for mutations using BLAT (UCSC Genome Browser).

### FACS analysis of monodispersed rat and mouse MECs

Rat and mouse mammary epithelial cells (RMECs and MMECs) were prepared from LN-excised abdominal and adjacent inguinal mammary glands, as described previously [25], [42]. For phenotyping RMECs and MMECs, staining was done using the low-volume staining method to reduce antibody costs [42]. To stain the single RMECs, antibodies against rat CD49f (Santa Cruz), CD24, CD29, CD31, CD45, and CD61 (BD Biosciences) or peanut lectin (Sigma) were used. To stain MMECs, antibodies against mouse CD24, CD29, CD31, CD45, CD49f, and CD61 (BD Biosciences) were used. Live cells were gated based on FSC, SSC, and Hoechst staining for 2n–4n DNA content. Single cells were gated using forward scatter (FSC) and side scatter (SSC) width. For phenotyping, the stained samples were acquired on a BD LSR II flow cytometer equipped with 4 lasers (multi-line UV, 405 nm, 488 nm and 633 nm). The data were collected as fcs3 files using FACS Diva software and analyzed using FlowJo software (Treestar Inc). Data files obtained from cell samples stained with single antibodies and control unstained cell samples were used for compensation. Data on percentages of cells in various gated populations or mean fluorescence intensities of entire populations were exported and statistically analyzed using Student's t-test.

For RMEC sorting, 50–70 million single cells were stained with anti-rat CD24, CD29, CD31, CD45, CD61 and peanut lectin at a concentration recommended by the vendor's specifications. Sorting was done on a BD FACSAria flow cytometer equipped with 5 lasers (multi-line UV, 405 nm, 488 nm, 540 nm and 640 nm). Cells were collected in 50% FBS.

### RMEC transplantation and matrigel assays

We have previously shown that the clonogenic cells assayed in our transplant system are capable of dividing and differentiating into morphologically and functionally normal mammary parenchyma [41]. For this RMEC transplantation assay, donor and recipients from the susceptible congenic control line WF.Cop and *Mcs1a* resistant congenic lines W4 and W5 were used. The final dilutions of single RMECs were mixed with an equal volume of a 50% brain homogenate, which was extracted from the donor rats. Aliquots of 40 µl of the mixture containing a known number of cells were then injected into the interscapular fat pad of recipient animals of the same genotype. The frequency of viable clonogenic stem-like cells in a cell suspension was quantitated using a limiting dilution assay as previously described [98], [99]. In each rat, two sites were used for grafting. For each cell dilution, between 8 and 32 sites were transplanted per genotype. Recipient rats were sacrificed 6 weeks after mammary cell grafting, and the fat pads injection sites were removed, fixed, stained, and examined for the growth of mammary tissue. The percentages of transplant sites with a mammary outgrowth were then plotted against the number of cells injected per site. The data were fit to the transplantation model of Porter et al. [100] and according to the statistical methodology for this model there was no significant difference between any of the 3 groups in the estimated number of cells required to give 50% outgrowth occurrence. For display purposes, the data for transplant sites from congenic lines W4 and W5 were combined and plotted in Figure 4A as a single line for the resistant genotype. A second statistical approach was taken to detect a possible difference in outgrowths at each cell number individually between the susceptible and resistant (W4 and W5 combined) genotypes. Therefore, Chi-square tests for independent distributions in a 2×2 contingency matrix were conducted for comparing susceptible to resistant for each cell number. The P-values were adjusted for multiple comparisons.

For the matrigel assay, single sorted RMEC suspensions containing 10,000 cells were spun 450× g for 5 minutes at 4°C. Supernatant was discarded, the cell pellet was resuspended in 100 uL phenol red free Matrigel (BD Biosciences) and immediately plated in 12- or 24-well plates while on ice. Plates were placed at 37°C (with 5% CO_2_) for 30–60 minutes to allow gelling process. Mammary Epithelial Cell Medium (PromoCell) with 5% bovine calf serum and antibiotics was then added to the wells containing the sorted cells in matrigel. Fresh media was provided on days 2 and 5. On day 10, Matrigel containing RMEC colonies was fixed with 2% paraformaldehyde in phosphate buffer pH 7.4 (PB) for 30 minutes at 37°C followed by staining with 0.5% methylene blue in PB for 5 minutes at 37°C. Colonies were counted using a microscope. Count data were normalized to the average colony count of the susceptible congenic control line run in the same experiment and was statistically analyzed using Student's t-test.

### Cell culture and transfection

MCF10A and MCF7 cell lines were obtained (ATCC) and cultivated according to the manufacturer's recommendations. Transient transfections were done in 24-well plates using the Lipofectamin2000 reagent (Invitrogen). Short interfering RNAs (*NR2F1* SMARTpool and Non-targeting pool) were obtained (Dharmacon) and used at a final concentration of 125 nM in the transfection media. The transfection media was washed off the cells after 6 hours. The cells were cultured for 40 additional hours before harvesting for FACS and expression analysis.

### *NR2F1* transcript levels in human breast cancer data sets

The Oncomine database (www.oncomine.org) was queried using the following filters: Gene: *NR2F1*, Cancer Type: Breast carcinoma, Sample Type: Clinical Specimen. Only studies with 120+ samples were considered. If available, the median levels of *NR2F1* for the clinical parameters, histological grade, ER-status, PR-status, HER2-status, TN-status were entered in Excel. To be able to compare clinical parameters across studies, the median levels for each clinical parameter in a study were normalized by the median level for the entire study. The average of the normalized median values was plotted. For statistical analysis, the non-parametric Kruskal-Wallis test was used.

To ask if lower *NR2F1* levels are associated with histological grade 3 in both ER-positive and ER-negative breast cancers or HER2-positive and HER2-negative breast cancers, the RMA normalized *NR2F1* probe set levels (probe ID 209505_at) from the GSE3494 and GSE5460 studies were used, respectively. To match the Y-axis scale in panel A, the RMA normalized values were expressed relative to the median level for the entire study. The values were statistically compared between groups using the non-parametric Mann-Whitney U test.

## Supporting Information

Figure S1 Southern blot analysis, genotyping and additional gene expression data on WT and MD mice. A) Image of Southern blot analysis of a correctly targeted ES cell clone (low and high DNA content in agarose gel lanes). Briefly, splits from individual clones were picked into 96 well plates, lysed and digested with *Mfe*I. Digests were run on an agarose gel, denatured with sodium hydroxide and transferred to Hybond membranes. Radioactively labeled probe (prepared by PCR of sequence: chr13:78,913,156–78,913,622, UCSC Genome Browser, version mm9) was hybridized, detecting a 9 Kb or 23 Kb fragment for the wild type or targeted allele, respectively. B) Image of genotyping PCR products on genomic DNA samples from mice wild type (WT), heterozygous (H), or homozygous (MD) for the megadeletion mutation. Each genomic DNA sample is analyzed by two PCR assays. On the left section of the gel image are shown the results of the PCR assay with primers that are located to the 3′ and 5′ MICER clone, respectively, spanning the deletion (mmMICERdel). On the right section of the gel image are shown the results of the PCR assay with primers that are located to sequences within the deleted region (mmdelNeg, all primers sequences in Methods). WT animals are defined by absence of the mmMICERdel band and presence of the mmdelNeg band. MD animals are identified by presence of the mmMICERdel band and absence of the mmdelNeg band. H animals are identified by presence of both bands. The DNA-ladder in the picture is a 100 bp ladder, with the 100 bp, 500 bp and 1,000 bp markers indicated. C) *Nr2f1* transcript levels in liver, ovary and thymus tissue. Graphed are the average (+/− sem) *Nr2f1* transcript levels normalized to the transcript level of the *ActB* endogenous control, relative to the average of the WT group. WT = wild type, MD = megadeletion. Liver: MD n = 7, WT n = 5; Ovary: MD n = 7, WT n = 5; Thymus: MD n = 7, WT n = 4. Significantly different (P<0.05) levels between WT and MD (FVB) samples are indicated with an asterisk.
(TIF)

Figure S2 A) Zoom-in plot of the *Mcs1a* region showing increased relative interaction frequency with the *Nr2f1* promoter fragment (adapted from Figure 3D). The locations of the *Bgl*II sites, 3C primers and polymorphisms are shown on a genomic map derived from version rn4 of the rat genome. B) The 4^th^ polymorphism is shown in detail and is predicted to change a rat-mouse-human conserved COUP-TF binding motif (V$Coup_01). C) Flow chart illustrating that the *Mcs1a* resistance allele harboring the 14 bp deletion of the COUP-TF binding motif upregulates *Nr2f1* transcript levels, which is associated with decreased mammary carcinoma multiplicity.
(TIF)

Figure S3 Matrigel colony-forming ability for luminal RMEC subpopulations. In the upper left panel, a representative FACS dot plot is shown for luminal RMECs labeled with anti-CD61 and peanut lectin (PNL). Three gates were applied to sort the CD61+PNLhi (green), the CD61hiPNL+ (orange) and the entire CD61+ (purple) populations of luminal RMECs. An equal number of cells was plated in Matrigel to test for colony-forming ability. In the lower right panel, the results of the colony-forming ability assay are shown as the average (+/− sem) number of colonies relative to the number of colonies for the CD61+ luminal population (n = 8 assays). The CD61+PNLhi population had a significantly increased and the CD61hiPNL+ had a significantly decreased colony-forming ability as compared with the CD61+ luminal population (P<0.05). For each sorted population, the range of absolute amount of colonies per 10,000 plated cells is printed in the lower left panel.
(TIF)

Figure S4 A) Graphed are the average (+/−sem) *NR2F1* transcript levels relative to transcript levels of the *GAPDH* endogenous control for siRNA-treated cell lines MCF10A and MCF7. B) Mean fluorescence intensities in artificial units (AU) of anti-CD24, -CD29, -CD49f and -CD44 labeling of MCF10A and MCF7 cells treated with siRNAs against *NR2F1* (siNR2F1) or non-targeting control siRNAs (siCONTROL). Significantly different mean fluorescence intensity (P<0.05) is indicated by an asterisk.
(TIF)

Table S1 Markers with asterisk designed for negative strand. Markers in bold used to define congenic lines in Figure 1. Markers in grey shading defining the current *Mcs1a* critical interval boundaries. gUwm, g2Uwm, bUwm are microsatelite markers. rf are SNP markers, discovered by resequencing rat-fugu (rf) conserved elements in the *Mcs1a* locus; variants are listed in Table S3.
(XLSX)

Table S2 Positions are in basepairs on rat chromosome 2 (UCSC version 3.4/rn4).
(XLSX)

Table S3 Positions are in basepairs on rat chromosome 2 (UCSC version 3.4/rn4). For all variants, the WF allele matches the genome sequence (from the Brown Norway inbred rat strain). All variants have been submitted to dbSNP (accession number pending).
(XLSX)

Table S4 Listed are 1,531 genes with a unadjusted P-value of <0.05, sorted by P-value starting with the 412 genes with 1-1-1 mouse-rat-human orthologs. LogFC = log of the Fold Change between the WT and MD samples. The WT.1-4 and MD.1-4 columns contain the RSEM output .nu values.
(XLSX)

Table S5 The same groups and background list were run using 2 online GO enrichment tools, GOrilla and DAVID. The enriched terms printed in red reach best significance, as determined by an adjusted P-value<0.05 (FDR adjust in GOrilla, Benjamini-Hochsberg adjust in DAVID).
(XLSX)

Table S6 The same groups and background list were run using 2 online GO enrichment tools, GOrilla and DAVID. The enriched terms printed in red reach best significance, as determined by an adjusted P-value<0.05 (FDR adjust in GOrilla, Benjamini-Hochsberg adjust in DAVID).
(XLSX)

Table S7 The same groups and background list were run using 2 online GO enrichment tools, GOrilla and DAVID. The enriched terms printed in red reach best significance, as determined by an adjusted P-value<0.05 (FDR adjust in GOrilla, Benjamini-Hochsberg adjust in DAVID).
(XLSX)
