# Supplementary figures and images for: The Gene Desert Mammary Carcinoma Susceptibility Locus Mcs1a Regulates Nr2f1 Modifying Mammary Epithelial Cell Differentiation and Proliferation
Source: PLoS Genet. 2013 Jun 13;9(6):e1003549. doi: 10.1371/journal.pgen.1003549 (PMC3681674; doi:10.1371/journal.pgen.1003549)

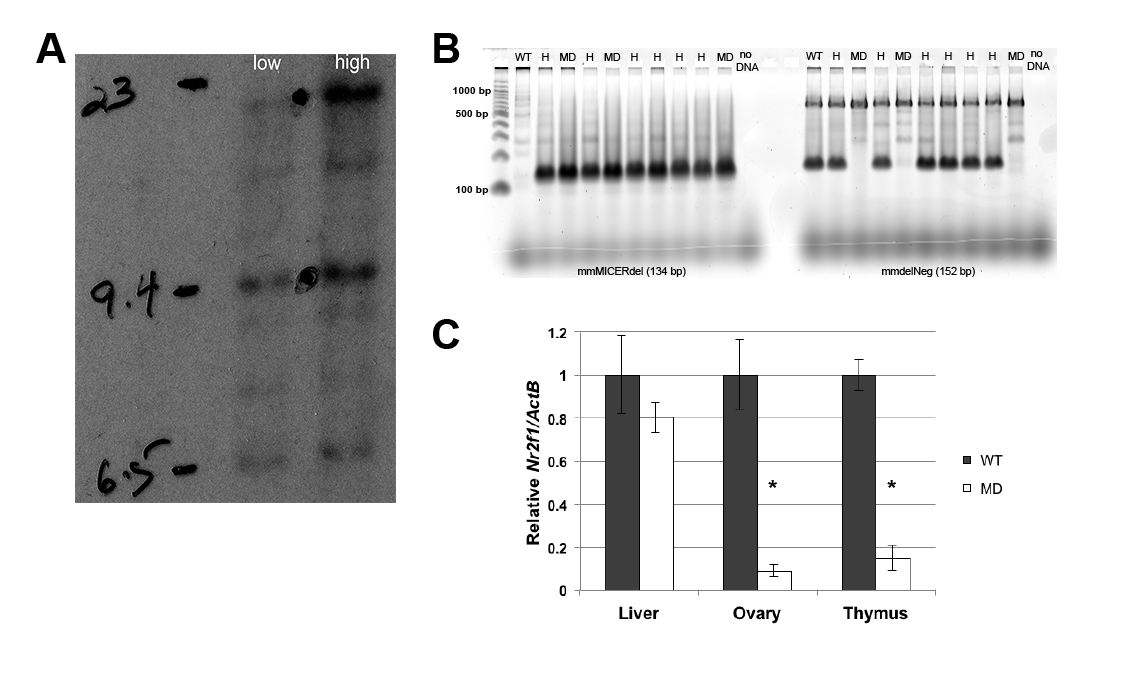

Supplement: Figure S1 — Southern blot analysis, genotyping and additional gene expression data on WT and MD mice. A) Image of Southern blot analysis of a correctly targeted ES cell clone (low and high DNA content in agarose gel lanes). Briefly, splits from individual clones were picked into 96 well plates, lysed and digested with MfeI. Digests were run on an agarose gel, denatured with sodium hydroxide and transferred to Hybond membranes. Radioactively labeled probe (prepared by PCR of sequence: chr13:78,913,156–78,913,622, UCSC Genome Browser, version mm9) was hybridized, detecting a 9 Kb or 23 Kb fragment for the wild type or targeted allele, respectively. B) Image of genotyping PCR products on genomic DNA samples from mice wild type (WT), heterozygous (H), or homozygous (MD) for the megadeletion mutation. Each genomic DNA sample is analyzed by two PCR assays. On the left section of the gel image are shown the results of the PCR assay with primers that are located to the 3′ and 5′ MICER clone, respectively, spanning the deletion (mmMICERdel). On the right section of the gel image are shown the results of the PCR assay with primers that are located to sequences within the deleted region (mmdelNeg, all primers sequences in Methods). WT animals are defined by absence of the mmMICERdel band and presence of the mmdelNeg band. MD animals are identified by presence of the mmMICERdel band and absence of the mmdelNeg band. H animals are identified by presence of both bands. The DNA-ladder in the picture is a 100 bp ladder, with the 100 bp, 500 bp and 1,000 bp markers indicated. C) Nr2f1 transcript levels in liver, ovary and thymus tissue. Graphed are the average (+/− sem) Nr2f1 transcript levels normalized to the transcript level of the ActB endogenous control, relative to the average of the WT group. WT = wild type, MD = megadeletion. Liver: MD n = 7, WT n = 5; Ovary: MD n = 7, WT n = 5; Thymus: MD n = 7, WT n = 4. Significantly different (P<0.05) levels between WT and MD (FVB) samples are indic [file pgen.1003549.s001.tif]

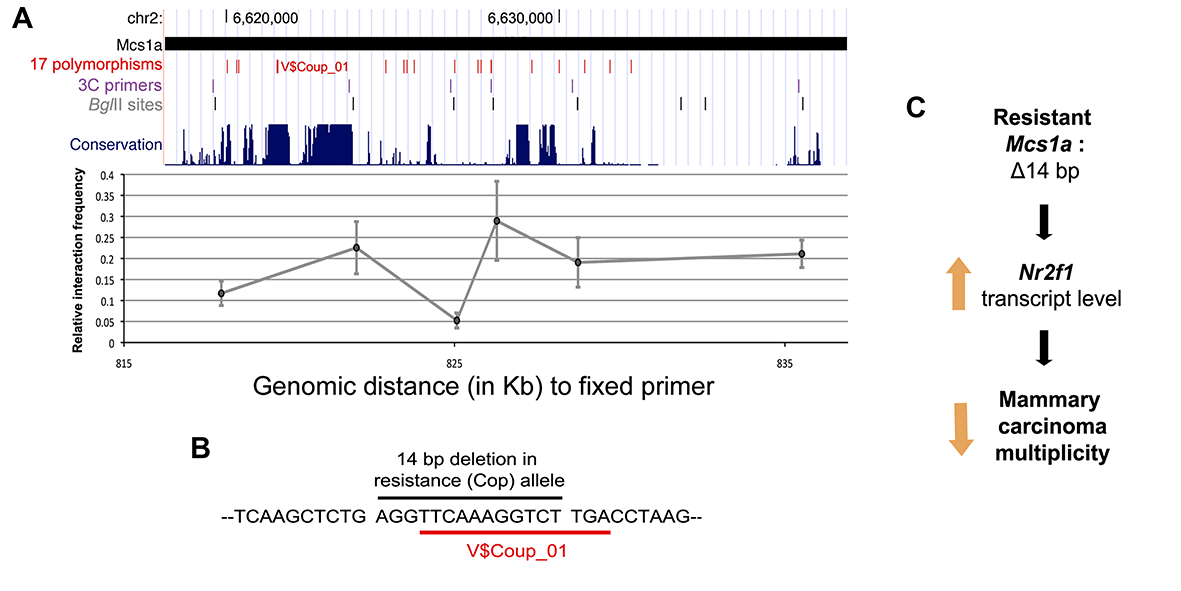

Supplement: Figure S2 — A) Zoom-in plot of the Mcs1a region showing increased relative interaction frequency with the Nr2f1 promoter fragment (adapted from Figure 3D). The locations of the BglII sites, 3C primers and polymorphisms are shown on a genomic map derived from version rn4 of the rat genome. B) The 4th polymorphism is shown in detail and is predicted to change a rat-mouse-human conserved COUP-TF binding motif (V$Coup_01). C) Flow chart illustrating that the Mcs1a resistance allele harboring the 14 bp deletion of the COUP-TF binding motif upregulates Nr2f1 transcript levels, which is associated with decreased mammary carcinoma multiplicity. (TIF) [file pgen.1003549.s002.tif]

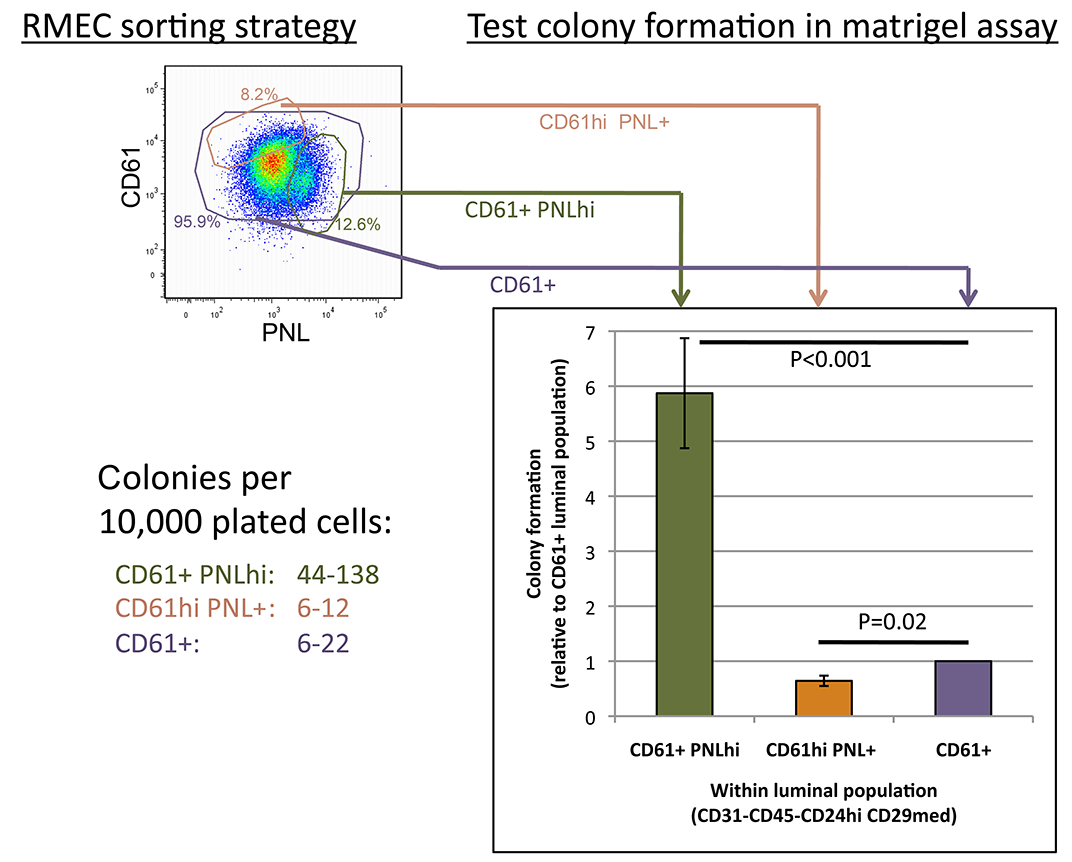

Supplement: Figure S3 — Matrigel colony-forming ability for luminal RMEC subpopulations. In the upper left panel, a representative FACS dot plot is shown for luminal RMECs labeled with anti-CD61 and peanut lectin (PNL). Three gates were applied to sort the CD61+PNLhi (green), the CD61hiPNL+ (orange) and the entire CD61+ (purple) populations of luminal RMECs. An equal number of cells was plated in Matrigel to test for colony-forming ability. In the lower right panel, the results of the colony-forming ability assay are shown as the average (+/− sem) number of colonies relative to the number of colonies for the CD61+ luminal population (n = 8 assays). The CD61+PNLhi population had a significantly increased and the CD61hiPNL+ had a significantly decreased colony-forming ability as compared with the CD61+ luminal population (P<0.05). For each sorted population, the range of absolute amount of colonies per 10,000 plated cells is printed in the lower left panel. (TIF) [file pgen.1003549.s003.tif]

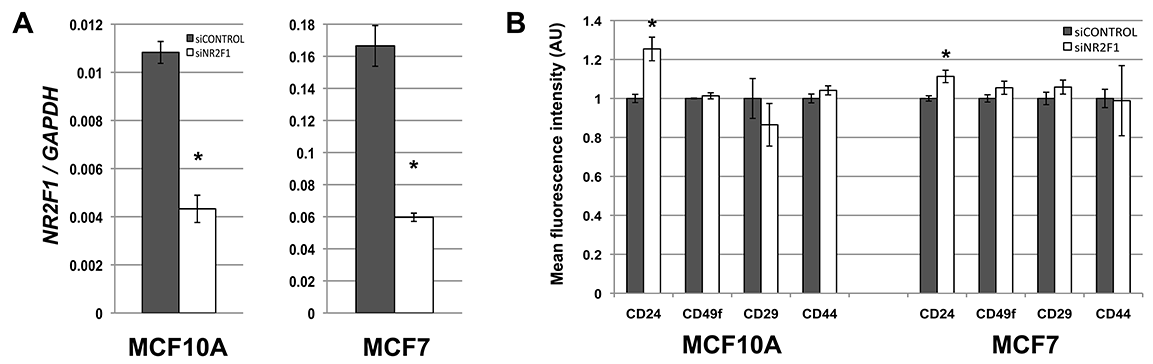

Supplement: Figure S4 — A) Graphed are the average (+/−sem) NR2F1 transcript levels relative to transcript levels of the GAPDH endogenous control for siRNA-treated cell lines MCF10A and MCF7. B) Mean fluorescence intensities in artificial units (AU) of anti-CD24, -CD29, -CD49f and -CD44 labeling of MCF10A and MCF7 cells treated with siRNAs against NR2F1 (siNR2F1) or non-targeting control siRNAs (siCONTROL). Significantly different mean fluorescence intensity (P<0.05) is indicated by an asterisk. (TIF) [file pgen.1003549.s004.tif]
